# An Improved Whale Optimization Algorithm via Angle Penalized Distance for Automatic Train Operation

**DOI:** 10.3390/biomimetics10060384

**Published:** 2025-06-09

**Authors:** Longda Wang, Yanjie Ju, Long Guo, Gang Liu, Chunlin Li, Yan Chen

**Affiliations:** 1 School of Electrical Engineering, Dalian Jiaotong University, Dalian 116023, China; wld@djtu.edu.cn; 2 School of Economics and Management, Gongqing Institute of Science and Technology, Jiujiang 332020, China; guo19820102@163.com; 3 College of Engineering, Inner Mongolia Minzu University, Tongliao 028000, China; liugang530242@imun.edu.cn; 4 Faculty of Vehicle Engineering and Mechanics, Dalian University of Technology, Dalian 116023, China; lichunlin@neusoft.com; 5 School of Mechanical and Electrical Engineering, Chizhou University, Chizhou 247000, China; chenyan2023@czu.edu.cn

**Keywords:** automatic train operation, target speed curve, multi-objective optimization, improved whale optimization algorithm

## Abstract

This study proposes a novel effective improved whale optimization algorithm via angle penalized distance (IWOA-APD) for automatic train operation (ATO) to effectively improve the ATO quality. Specifically, aiming at the high-quality target speed curve of urban rail trains, a target speed curve multi-objective optimization model for ATO is established with energy saving, punctuality, accurate stopping, and comfort as the indexes; and the comprehensive evaluation strategy utilizing angle-penalized distance as the evaluation index is proposed to enhance the assessment’s rationality and applicability. On this basis, the IWOA-APD is proposed using strategies of non-linear decreasing convergence factor, solutions of out-of-bounds eliminating via combination of reflection and refraction, mechanisms of genetic evolution with variable probability, and elite maintenance based on fusion distance and crowding degree distance. In addition, the detailed design scheme of IWOA-APD is given. The test results show that the proposed IWOA-APD achieves significant performance improvements compared to traditional MOWOA. In the optimization scenario from Lvshun New Port Station to Tieshan Town Station of Dalian urban rail transit line No.12, the IGD value shows a remarkable 69.1% reduction, while energy consumption decreases by 12.5%. The system achieves a 64.6% improvement in punctuality and a 76.5% enhancement in parking accuracy. Additionally, comfort level improves by 15.9%.

## 1. Introduction

Urban rail train is widely developed in public transit industry, as it has the superiorities of safety, speed, punctuality, low consumption, environmental friendliness, convenience, and large carrying capacity. The ATO system is one of significant applications of urban rail trains, and its multi-objective comprehensive performances comprising evaluation indexes for safety, energy saving, comfort, punctuality, etc., are guaranteed by its subsystem, the automatic train operation control (ATOC) system [1]. During the whole process of ATOC of urban rail train, target speed curve optimization and tracking control are two essential segments. Target speed curve is the tracking object of the ATOC system, and obtaining an ideal optimum curve is crucial for the safe and comfortable operation, energy saving, consumption reduction, and precise parking of ATO [2]. If the efficient optimum target speed curve cannot be obtained, even with stable, fast, and accurate ATO tracking control technology, the final comprehensive performance quality of the ATO will not be perfect [3]. To achieve efficient target speed curve optimization, it is necessary to explore effective target speed curve optimization models and algorithms for ATO.

Research in the field of ATO optimization can be traced back to the 1960s. At that time, the relevant general calculators were developed by American Railways. Khmelnitsky established the one-size-fits-all rules of operation sequence for train operation optimization [4]. For reducing the energy consumption of train operation process, Chang proposed a method for optimizing the position sequence of train idling points based on a genetic algorithm [5].

At present, the research about target speed curve optimization for ATO has become a research focus in the urban rail transit field. Considering the balance between energy saving and rapid operation, an improved particle swarm optimization algorithm for target velocity trajectory optimization of an urban rail train was proposed [6]. To obtain satisfactory optimization results, based on a train speed trajectory multi-objective optimization model, an improved optimization algorithm combining differential evolution and simulated annealing was proposed [7]. A novel model for energy-efficient ATO was established, and the method of combining analytical and numerical methods for deriving energy-efficient train operation strategies was also given [8]. To reduce energy consumption and arrival delay for the later journey of the train, a multiple-optimization model based on energy-efficient operation method was proposed and verified [9]. An intelligent metro ATO optimization expert system, based on extensive ATO data collection and a comprehensive heuristic expert reasoning method, was developed, and the optimization simulation test results of Beijing Metro Yizhuang line showed that compared with the traditional ATO optimization system, the developed intelligent metro train operation optimization expert system has stronger optimization ability, and it can find the train operation strategy with less running time error and energy consumption [10]. For target speed curve optimization of urban rail train, considering driving safety and reducing energy consumption of train operation process, based on the collected ATO data, a series of intelligent safe driving methods were applied, which can obtain more desirable results [11]. It can be seen from the above research that a series of advanced and intelligent elements based on driving experience, massive ATO process data, expert system, and so on, are also deeply integrated into the establishment of the target speed curve optimization model of the urban rail train, and various traditional optimization algorithms, such as genetic algorithm, particle swarm optimization and differential evolution, have a wide range of applications in the research and implementation of target speed curve optimization for ATO.

However, traditional optimization algorithms often fall into local optima, which poses several problems, especially in the late stage of evolution. The three most significant aspects include local convergence, inefficient searching, and slow optimization speed. Compared with traditional optimization algorithms, the whale optimization algorithm (WOA) has an efficient optimization mechanism, so its global optimization search ability is very strong, even in the late stage of evolution. The whale optimization algorithm (WOA) is fundamentally rooted in bionics principles, specifically mimicking the unique cooperative hunting behaviors of humpback whales. This bionic foundation not only provides WOA with superior optimization capabilities but also offers interpretable insights into its working principles, distinguishing it from other optimization approaches. For improving the global optimization performance of WOA, extensive research has proposed some improvement strategies. To solve the problems of WOA with slow convergence speed and low accuracy, a multistrategy hybrid adaptive whale optimization algorithm (MHWOA) was proposed [12]. In addition, a hybrid whale optimization algorithm based on symbiotic strategy (HWOAMS) was proposed in [13], a binary whale optimization algorithm (BWOA) based on two transfer functions (the S-shaped and V-shaped) in [14], a deep-chaotic nature whale optimization algorithm using machine and deep learning (DL) models in [15], a new-type WOA based on the Q-learning algorithm and the exponential monte carlo acceptance probability (QWOA-EMC) in [16], and a novel enhanced whale optimization algorithm integrated with salp swarm algorithm ESSAWOA was also suggested. The main idea of ESSAWOA is to enhance the whale optimization algorithm (WOA) by combining the mechanism of the salp swarm algorithm (SSA) and the lens opposition-based learning strategy (LOBL) [17]. The aforementioned studies can promote the optimization capability of WOA.

The WOA can effectively improve target speed curve optimization performance for ATO. However, the effective comprehensive evaluation strategy via angle penalized distance (APD) for ATO has not been taken into account, thus leading to a deficiency in both the rationality and practical applicability of the assessment. Consequently, this impacts the effectiveness of optimizing the target velocity curve. In addition, traditional improved whale optimization algorithms do not fully consider the effective balance of global and local searches, and this affects its improvement effect to some extent. Hence, further improvements are necessary. An IWOA via angle penalized distance is proposed in this study, and strategies of non-linear decreasing convergence factor, solutions of out-of-bounds eliminating via combination of reflection and refraction, mechanisms of genetic evolution with variable probability, elite maintenance based on fusion distance, and crowding degree distance are also integrated into its computing frame so as to improve global optimization performance significantly. For testing the effect of the proposed algorithm, a hardware-in-the-loop (HIL) experimental platform for target speed curve optimization and tracking control for ATO was established in this study. Under the scenarios from Lvshun New Port to Tieshan Town pertaining to urban rail transit line No.12, from Jiu Li to 19th Bureau pertaining to the second-phase project of urban rail transit line No.13 in Dalian, more satisfactory target speed curve optimization and tracking control results for ATO can be obtained using the IWOA-APD. The IWOA-APD applied to target speed curve optimization for ATO proposed in this study has the following three contributions.

(I) This study presents an effective target speed curve multi-objective optimization model for ATO: in the traditional whole-process optimization for ATO, train operation sequence and its position sequence are the decision variables, and accustomed minimum scales are 1 m or 0.1 s; however, for ATOC, the operation control switching points cannot be exactly equal to the operation changing point; the empirical system control period is taken from 10 ms to 50 ms; consequently, using a traditional train operation optimization model is coarse and mismatched. In this study, for establishing a target speed curve multi-objective optimization model for ATO, the operation control switching points sequence is used as a decision variable so as to provide convenience for the actual tracking control and further effectively improve the comprehensive performance of urban rail train tracking control.

(II) This study designs an effective comprehensive evaluation strategy via angle penalized distance (APD) for ATO: traditional evaluation indexes or strategies have disadvantages of inaccuracy, opacity, and subjectivity; to improve the reasonableness and applicability of performance evaluation, based on APD, which takes into account both convergence rate and population distribution, and combined with Pareto superior relationship and optimization quality score value, this study designs an effective comprehensive evaluation strategy via angle penalized distance for ATO, thus effectively improving reasonable evaluation and applicability.

(III) This study proposes an effective improved WOA: in the traditional improved whale optimization algorithm, it is difficult to design an improved strategy and mechanism to effectively balance the global and local search, so that it is easy to fall into the serious problem of local convergence. In this study, for computing frame design of improved WOA, strategies of non-linear decreasing convergence factor, solutions of out-of-bounds eliminating via combination of reflection and refraction, mechanisms of genetic evolution with variable probability, elite maintenance based on fusion distance, and crowding degree distance are rational designed and applied, thereby effectively improving the global optimization performance.

This paper is structured as follows: Section 2 presents an introduction to the effective target speed curve multi-objective optimization model for ATO; Section 3 illustrates the effective comprehensive evaluation strategy via APD for target speed curve optimization of urban rail train; Section 4 proposes an effective IWOA-APD for target speed curve optimization; and Section 5 presents the simulated and experimental outcomes and performs the corresponding analysis. Finally, Section 6 sums up this study.

## 2. Establishment of Constrained Multi-Objective Optimization Model

### 2.1. Constraints and Performance Indexes of ATO

Based on the force analysis during the whole process for ATO and Newton’s second law, the dynamic equation of urban rail ATO can be given. The specific dynamics equation is as follows:

(1) $$\begin{array}{c} \left\{\begin{array}{c} \frac{dx}{dt}=v\\ Mv\frac{dv}{dx}=f\left(u,v\right)-w\left(x,v\right) \end{array}\right. \end{array}$$

where *x* is the actual operating position of the train; *t* is the actual operating time of the train; *v* is the actual operating speed of the train; f(u,v) represents the self-generated force of the urban rail train, which could either be traction force or braking force; w(x,v) represents the additional resistance encountered during urban rail ATO.

(2) $$\begin{array}{c} M=\left(1+\gamma \right)\times m \end{array}$$

where *M* is the inertial mass of the train; γ represents the rotating mass coefficient of the train; and *m* represents the actual mass of the train.

During traction, f(u,v) is determined collectively by the traction characteristics of the urban rail train, input control parameters, and the train’s operating speed; whereas during braking, f(u,v) is defined by the braking characteristics of the urban rail train, input control parameters, and the operating speed, and *u* represents the input control quantity of the train [18].

During the whole process of ATO [19], various constraints need to be considered, such as speed limit, acceleration and impact rate, boundary conditions, and manipulation modes. The specific constraints are illustrated as follows.

(I) Speed limit

Safety is the primary consideration in ATO. Speeding is the main cause of safety risks. During the whole process for ATO in urban rail transit, the actual speed of the current position should not exceed the speed limit.

(3) $$\begin{array}{c} 0\leq v\left(x\right)\leq v_{\lim}\left(x\right) \end{array}$$

where $v\left(x\right)$ and $v_{\lim}\left(x\right)$ represent, respectively, the actual speed and the speed limit at the position point *x*.

(II) Acceleration and impact rate

To maintain operation comfort, the absolute value of acceleration/deceleration and impact rate (absolute value of acceleration change rate) at any time point during the whole process of ATO should not be less than the allowable maximum absolute value of acceleration and maximum impact rate.

(4) $$\begin{array}{c} \left|a\left(t\right)\right|\leq {\left|a\right|}_{\max} \end{array}$$

(5) $$\begin{array}{c} Ir\left(t\right)\leq Ir_{\max} \end{array}$$

where a(t) represents the absolute value of the acceleration/deceleration of the train at time point *t*; ${\left|a\right|}_{\max}$ represents the allowable maximum absolute value of acceleration; Ir(t) represents the train impact rate at time point *t*, and $Ir_{\max}$ represents the allowable maximum impact rate.

(III) Boundary conditions

The speed and position of the initial state for ATO are both 0, and the final speed at the terminal station is also 0, all of which should not exceed the allowed maximum parking error and punctuality error during the train operation process.

(6) $$\begin{array}{c} \left\{\begin{array}{c} t(0)=0,v(0)=v(X)=0\\ \left|t\left(X\right)-\bar{T}\right|\leq \Delta T_{\max},\left|X-\bar{D}\right|\leq \Delta S_{\max} \end{array}\right. \end{array}$$

where $\bar{T}$ is the expected time of ATO; $\bar{D}$ is the expected length of ATO; *X* is the actual operation length of ATO; $\Delta T_{\max}$ is the allowable maximum punctuality error; $\Delta S_{\max}$ is the allowable maximum parking error; t(x) represents the actual time at position point *x*.

(IV) Operation mode

The operation sequence of ATO is composed of operation working condition sequence UC and its transition point position sequence PC, where $UC=\left\{uc_{1},uc_{2},\ldots uc_{s}\right\}$, $PC=\left\{pc_{1},pc_{2},\ldots pc_{s}\right\}$, $0<pc_{1}<pc_{2}<\ldots <pc_{is}<\ldots <pc_{s}<X$, is and *s* are the operation working condition index and operation sequence length, respectively. During the whole process of urban rail trains, there are four primary states: maximum traction, constant-speed operation, coasting, and maximum braking. In the coasting state, no force is applied to the train, while during constant-speed operation, to counteract external resistance and achieve zero net acceleration, it is further divided into traction-based constant speed and braking-based constant speed. According to the traction calculation standards applicable in China, integers 1, 0, and −1 are typically used to represent maximum traction, coasting, and maximum braking, respectively. For traction-based and braking-based constant-speed states, the train must apply partial traction or braking forces precisely balancing the running resistance, denoted in this paper as tc and tb, with $tc\in \left(0,1\right)$, and $tb\in \left(-1,0\right)$.

(7) $$\begin{array}{c} uc_{is}\times uc_{is+1}\neq -1 \end{array}$$

where uc represents the set of operational states per unit time and $uc_{is}$ denotes the isth element of uc, which corresponds to the operational state of the urban rail train during the isth state cycle. This paper uses 1, tc, 0, tb, and −1 to correspond to the following five states: maximum traction, traction-based constant speed, coasting, braking-based constant speed, and maximum braking. Equation (7) indicates that for safety and passenger comfort, a traction state cannot directly transition to a braking state; it must pass through a coasting or constant-speed state as an intermediate phase.

Based on safety and comfort considerations, the distance between adjacent operating working condition transition points must be greater than the allowable minimum distance.

(8) $$\begin{array}{c} pc_{is+1}-pc_{is}>\Delta pc_{\min} \end{array}$$

where $\Delta pc_{\min}$ is the allowable minimum distance between adjacent operating working condition transition points.

The optimization of performance indexes such as energy saving, punctuality, parking accuracy and comfort, etc., should be taken into consideration during whole process of ATO, and the specific objective functions are described as follows.

(I) Energy saving

During the train operation process, the work performed to overcome the resistance and the additional energy consumption is called the energy consumption *E* of the train, and the specific calculation expression is as follows:

(9) $$\begin{array}{c} E=\int_{0}^{t\left(X\right)}\mu (t)dt+E_{A} \end{array}$$

where $E_{A}$ is the additional energy consumption during train operation process and μ(t) is the traction power of the train to overcome resistance at time point *t*.

(II) Comfort level

Comfort level quantifies the ride quality of passengers, and it is an important index to measure the comfort level of train operation. It is the absolute value of acceleration change per unit distance or per unit time, and its specific calculation expression is as follows:

(10) $$\begin{array}{c} K_{Jerk}=\frac{\sum_{i=1}^{ns}\left|a_{i}-a_{i-1}\right|}{\bar{D}} \end{array}$$

where $K_{Jerk}$ is comfort level; $a_{i}$ is the acceleration of the train at the *i*th working condition point; ns is the number of working condition points.

(III) Parking accuracy

Parking error refers to the absolute value of the difference between the actual operating distance and the expected operating distance during train operation process, and its specific calculation expression is as follows:

(11) $$\begin{array}{c} \Delta S=\left|\bar{D}-X\right|\leq \Delta S_{\max} \end{array}$$

where ΔS is the absolute value of parking error.

(IV) Punctuality

The punctuality error can be expressed as the absolute value of the difference between the actual operating time and the expected time during the train operation process, and its specific calculation expression is as follows:

(12) $$\begin{array}{c} \Delta T=\left|\bar{T}-t\left(X\right)\right|\leq \Delta T_{\max} \end{array}$$

where ΔT is the absolute value of the punctuality error.

According to the performance indexes such as energy conservation, comfort, parking accuracy and punctuality, etc., the specific optimization performance evaluation model of whole process of ATO is given as follows:

(13) $$\begin{array}{c} \min\left\{E,K_{Jerk},\Delta T,\Delta S\right\} \end{array}$$

It is necessary to make energy consumption, comfort level, parking error, and punctuality error as small as possible.

### 2.2. Target Speed Curve Multi-Objective Optimization Model for ATO

It is crucial to present the optimal target speed curve for ATO that meets several constraints and highly optimized based on multiple objectives such as energy consumption, comfort, parking error and punctuality error, etc., which is one of the prerequisites for realizing high-performance ATO.

For the target speed curve optimization, six constraints such as the dynamic equation, speed limit, resistance, absolute value of acceleration, operation mode conversion, and boundary conditions of urban rail trains must be considered [20].

(I) The speed at any point should not exceed the tracking control speed limit to avoid unsafe hidden dangers caused by over speed.

(II) In the ATOC model of urban rail train, the resistance of urban rail train must include both basic resistance and line additional resistance.

(III) The absolute value of acceleration and deceleration at any position should not exceed the allowable maximum absolute value by regulation.

(IV) The operation states for traction and braking cannot be directly converted.

(V) The speed value of the starting and ending positions is 0, and the initial time is also 0.

(VI) The absolute value of parking error ΔS and the absolute value of punctuality error ΔT should not be greater than the maximum allowable absolute value of parking error $\Delta S_{\max}$ and punctuality error $\Delta T_{\max}$.

Taking the energy consumption, comfort level, parking error, and punctuality error of four performance indexes as optimization objectives and taking into account the above six constraints, the target speed curve optimization model of urban rail trains can be described as follows:

(14) $$\begin{array}{c} \begin{array}{c} \min\;\left\{E,K_{Jerk},\Delta T,\Delta S\right\}\\ ST.\;\left\{\begin{array}{c} \frac{dx}{dt}=v\\ Mv\frac{dv}{dx}=f\left(u,v\right)-R\left(x,v\right)-b\left(u,v\right)\\ 0\leq v\left(x\right)\leq v_{\lim}\left(x\right)-v_{p}\\ R\left(v,x\right)=r_{0}\left(v\right)+R_{e}\left(x\right)\\ \left|a\left(k\right)\right|\leq {\left|a\right|}_{\max}\\ oc\left(k\right)\times oc\left(k+1\right)\neq -1\\ pc_{is+1}-pc_{is}>\Delta pc_{\min}\\ v\left(0\right)=t\left(0\right)=v\left(X\right)=0\\ \left|X-\bar{D}\right|<\Delta s_{\max}\\ \left|t\left(X\right)-\bar{T}\right|<\Delta T_{\max} \end{array}\right. \end{array} \end{array}$$

where $v_{p}$ is the protection speed to prevent over speed phenomenon caused by control speed overshoot; oc(k) represents the operating condition at the *k*th time cycle.

## 3. Design of Comprehensive Evaluation Strategy

### 3.1. Multi-Objective Optimization and Its Pareto Optimal Solution

The multi-objective optimization problem can usually be expressed as

(15) $$\begin{array}{c} \begin{array}{c} \min\;F\left(Dx\right)={\left(f_{1}\left(Dx\right),f_{2}\left(Dx\right),\ldots ,f_{n}\left(Dx\right)\right)}^{T}\\ \;s.t.\;g_{ig}\left(Dx\right)\leq 0,\;ig=1,2,\ldots ,ng \end{array} \end{array}$$

where Dx is the *d*-dimensional decision variable; $g_{ig}\left(Dx\right)$ is the igth equality or inequality constraint; ng is the number of constraint functions; and F(Dx) is the objective vector composed of *n* objective functions.

(16) $$\begin{array}{c} \begin{array}{c} Dx=(Dx^{1},Dx^{2},\ldots ,Dx^{d})\in \Omega \end{array} \end{array}$$

where Ω is the feasible solution space of the decision variable.

For multi-objective optimization problems, there is generally no optimal solution in the practical sense, but there are often many or even infinitely many mutually non-inferior Pareto optimal solutions [21].

When dealing with multi-objective optimization problems, the objectives are often contradictory, and it is difficult to find a solution to minimize all objective functions at the same time. Hence, when solving multi-objective problems, it is usual to find a group of balanced solutions for all objectives, that is, Pareto optimal solutions.

**Definition** **1** (**Pareto domination**)**.** *A solution Dx is said to dominate another solution Dy (denoted as Dx≺Dy) if and only if no component of Dy is smaller than the corresponding component of Dx and at least one component of Dx is strictly smaller, that is,*

(17) $$\begin{array}{c} \forall i\in \left\{1,2,...,m\right\}:f_{i}\left(Dx\right)\leq f_{i}\left(Dy\right)\wedge \;\exists j\in \left\{1,2,...,m\right\}:f_{j}\left(Dx\right)<f_{j}\left(Dy\right)\; \end{array}$$

*where m is the number of objectives [22].*

**Definition** **2** (**Pareto optimal solution set**)**.** *If any variable that dominates decision variable Dx cannot be found in the feasible solution space* Ω *of the decision variable, then the decision variable Dx is called a Pareto optimal solution. The set of all Pareto optimal solutions is the Pareto optimal solution set PS, and its expression is as follows:*

(18) $$\begin{array}{c} PS=\{Dx\in \Omega |\neg \exists Dy\in \Omega ,\;f(Dy)≺f(Dx)\} \end{array}$$

**Definition** **3** (**Pareto Frontier**)**.** *The graph mapped by all Pareto optimal solutions in the objective space becomes the Pareto Frontier, and its expression is detailed as follows:*

(19) $$\begin{array}{c} PF=\{F(Dx)|Dx\in PS\} \end{array}$$

Inverse Generation Distance (IGD) can be used as the evaluation index of the multi-objective optimization algorithm. The calculation formula of Inverse Generation Distance (IGD) is as follows:

(20) $$\begin{array}{c} IGD=\frac{\sum_{i=1}^{\left|P\right|}d\left(P_{i},P^{\prime }\right)}{\left|P\right|} \end{array}$$

where *P* is the set of uniform sampling points at the real Pareto Frontier; $P^{\prime }$ is the approximate Pareto solution set obtained by the algorithm to be tested; |P| is the scale of the set *P*; and $d(P_{i},P^{\prime })$ is the minimum distance between the *i*th Pareto sampling point $P_{i}$ and the approximate Pareto solution set $P^{\prime }$.

Accordingly, the IGD value is lower, the convergence of the Pareto solution set obtained by the algorithm is better and closer to the true Pareto front.

### 3.2. Linear Weighting Method

Compared with the multi-objective optimization problem, the single-objective optimization problem is easier to solve. The unified index is to establish a new optimization objective to replace the original multiple optimization objectives so as to achieve the purpose of transforming the original multi-objective optimization problem into a single objective optimization problem [23]. Linear weighting method is a commonly used unified index method. In order to eliminate the adverse effects of the dimensional and order of magnitude differences of each optimization objective on the optimization results, data normalization processing is required.

The calculation formula for normalized linear weighting objective can be expressed as follows:

(21) $$\begin{array}{c} \left\{\begin{array}{c} F^{\prime }\left(x\right)=\sum_{i=1}^{k}{\omega^{\prime }}_{i}{\omega^{\prime \prime }}_{i}f_{i}\left(x\right)\\ {\omega^{\prime \prime }}_{i}=\frac{f_{i}\left(x\right)-\min\left(f_{i}\left(x\right)\right)}{\max\left(f_{i}\left(x\right)\right)-\min\left(f_{i}\left(x\right)\right)} \end{array}\right. \end{array}$$

where $\omega^{\prime }$ is the characteristic weight factor; $\omega^{\prime \prime }$ is the correction weight factor which is used to eliminate the influence caused by the difference in dimension and magnitude of the optimization objective, and min and max are the minimum and maximum values of a function, respectively.

(22) $$\begin{array}{c} \sum_{i=1}^{m}{\omega^{\prime }}_{i}=1 \end{array}$$

where the formula meets and reflects the relative importance of the optimization objective.

Compared with Pareto dominance, the unified index can clearly evaluate the advantages and disadvantages of any different decision variables. However, the value of characteristic and correction weight factor $\omega^{\prime }$ and $\omega^{\prime \prime }$ lack specific theoretical basis, so this method has certain subjective limitations.

### 3.3. Angle Penalty Distance

In the iterative calculation process of multi-objective optimization algorithm, the decision variables that need to be evaluated are usually mutually non-inferior. Only using Pareto domination as the evaluation standard makes it difficult to choose most of the decision variables so that the optimization efficiency is sharply reduced, which is not conducive to finding the ideal optimization solution. However, using the traditional linear weighting method is far from the objective reality [24].

Generally, the two-norm distance is widely used, and the calculation formula of the distance of the objective weight vector F(x) is as follows:

(23) $$\begin{array}{c} d\left(x\right)={∥F(x)∥}_{2}=\sqrt{\sum_{i=1}^{m}f_{i}{\left(x\right)}^{2}} \end{array}$$

where $f_{i}\left(x\right)$ is the normalized value of the *i*th objective for the decision variable *x*.

The traditional unification index is usually based only on distance and does not consider the angle. For decision variable *x*, the minimum angle θ(x) between it and other individuals in the population *P* can be used to measure its distribution.

The specific minimum angle θ(x) can be calculated as follows:

(24) $$\begin{array}{c} \begin{array}{c} \theta \left(x\right)=\min_{y\in p,y\neq x}\mathrm{angle}\left(x,y\right)\\ \mathrm{angle}\left(x,y\right)=\arccos\left(\frac{F\left(x\right)F\left(y\right)}{{∥F\left(x\right)∥}_{2}{∥F\left(y\right)∥}_{2}}\right) \end{array} \end{array}$$

A larger minimum angle θ(x) indicates a better distribution of the decision variable *x* in the population.

Angle penalized distance (APD) is a dual metric that simultaneously considers both convergence and distribution. As an effective indicator for dynamically balancing the relative importance of convergence and distribution during multi-objective evolutionary processes, APD plays a crucial role in our experimental framework.

The specific calculation method of the improved APD is as follows:

(25) $$\begin{array}{c} \begin{array}{c} APD\left(x\right)=\left(1+P(\theta )\right)d\left(x\right)\\ P\left(\theta \right)=M_{P}{t_{r}}^{\alpha_{P}}\cos\left(\theta (x)\right)\alpha_{P} \end{array} \end{array}$$

where P(θ) is the penalty factor; $\alpha_{P}$ is the variable rate factor, which is used to control the increasing trend of the importance of distribution; $M_{P}$ is the penalty coefficient, and it is usually taken as the value of the target dimension, that is, $M_{P}=m$.

(26) $$\begin{array}{c} t_{r}=\frac{t}{T_{\max}} \end{array}$$

where $t_{r}$ is the evolution process; *t* and $T_{\max}$ are the current number of iterations and the maximum number of iterations, respectively.

**Figure 1:**
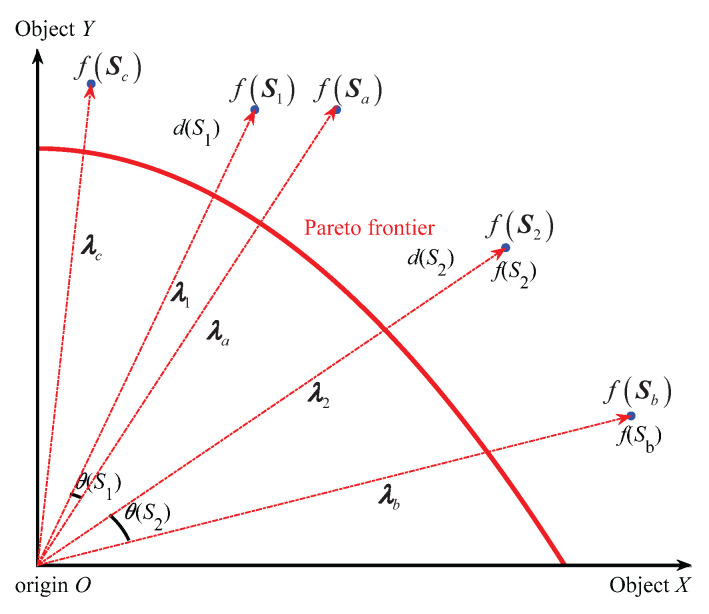
Schematic diagram of evaluating the quality relationship between solutions using angle penalty distance (APD) in the early stage of evolution.

**Figure 2:**
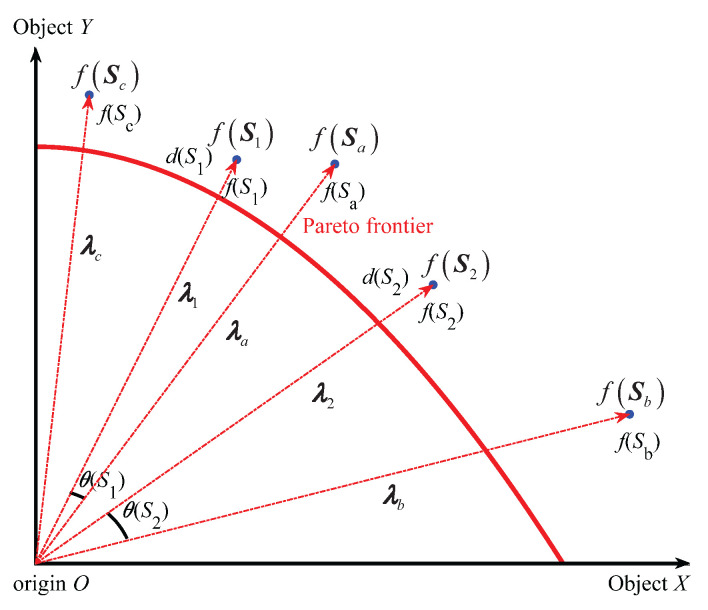
Schematic diagram of evaluating the quality relationship between solutions using angle penalty distance (APD) in the late stage of evolution.

The specific schematic diagram of evaluating the quality of solutions by (APD) in the early and late evolution stages is shown in Figure 1 and Figure 2 below.

In Figure 1 and Figure 2, the two solid line coordinate axes represent two optimization objectives, $S_{1}$, $S_{2}$, $S_{a}$, $S_{b}$ and $S_{c}$ are the five mutually non-inferior solutions in the population, the solution objective functions are $f(S_{1})$, $f(S_{2})$, $f(S_{a})$, $f(S_{b})$ and $f(S_{c}t)$, respectively, and are represented by blue solid circles. $S_{1}$ and $S_{2}$, respectively, represent the solutions to be compared, with distances of $d(S_{1})$ and $d(S_{2})$, respectively. $S_{a}$ and $S_{b}$ represent the solution in the population that has the smallest angle with $S_{1}$ and $S_{2}$, with the minimum angles being $\theta (S_{1})$ and $\theta (S_{2})$, respectively. Five red dashed lines with arrows represent the objective vectors $\lambda_{1}$, $\lambda_{2}$, $\lambda_{a}$, $\lambda_{b}$, $\lambda_{c}$ of the solutions $S_{1}$, $S_{2}$, $S_{a}$, $S_{b}$ and $S_{c}$. The red arc indicates the Pareto frontier.

According to Figure 1, it can be seen that in the early stage of iteration, each individual is far from the Pareto frontier, so evolution should focus on convergence. Since the current iteration number *t* is very small, the evolution process $t_{r}$ is close to 0, making the penalty factor P(θ) small, and thus APD(x) close to the distance d(x). In this case, $S_{1}$ is superior to $S_{2}$, because it is closer to the Pareto front ($d\left(S_{1}\right)<d\left(S_{2}\right)$). According to Figure 2, it can be seen that in the late stage of iteration, the entire population tends to approach the Pareto frontier, so evolution should focus on distribution. Since the current iteration number *t* is close to the maximum iteration number $T_{max}$, and the evolution process $t_{r}$ is close to 1, the penalty factor P(θ) is very large; hence, APD(x) is mainly determined by the minimum angle θ(x). In this case, $S_{2}$ is superior to $S_{1}$, because it has better distribution property ($\cos\left(\theta (S_{2})\right)<\cos\left(\left(S_{1}\right)\right)$).

Accordingly, it can be seen that the APD takes into account both convergence and distribution, and as an evaluation index for solution quality, it can effectively balance the importance of convergence and distribution in the evolutionary process.

### 3.4. Comprehensive Evaluation Strategy

In the target speed curve optimization for ATO, for the evaluation of the solution vector, the higher the optimization quality evaluation level of its performance index, the more favored it is by decision-makers. The classification of quality evaluation levels for ATO of Dalian urban rail transit line 12 and line 13 is shown in Table 1.

In Table 1, “excellent”, “medium”, and “poor” are the evaluation levels of the optimization quality, and their score values are set to 3, 2, and 1, respectively. Obviously, the highest score value of the optimization quality of the solution vector is 12; $E_{0}$, $E_{1}$, and $E_{2}$ are the boundary values of the optimization quality evaluation level of energy consumption, respectively; +∞ is a large enough positive real number; the boundary values of the preference evaluation level for punctuality, parking accuracy, and comfort level should refer to the relevant provisions of the international standard ISO2631, and the decision-maker should offer a more objective value according to the actual operation situation.

In the calculation process of target speed curve optimization for ATO, for any two solution vectors, the following steps are used to evaluate the quality of the solutions.

(I) If there is a Pareto superior dominant relationship between the two solution vectors, the Pareto superior solution vector is better; otherwise, the next step of evaluation is carried out.

(II) If the optimization quality score values between the two solution vectors are different, the solution vector with higher score value is better; otherwise, the next step of evaluation is carried out.

(III) Comparing the APD of the two solution vectors, the solution vector with smaller APD is better. Since the angular penalty distance is a real number, there is a clear size relationship.

It can be seen from the above evaluation strategy given in this study that the evaluation criterion of the quality of the solution takes into account the APD, that is, the evaluation process not only considers the objective reality of the optimization problem, but also considers the expectations of the decision-maker based on the actual situation.

## 4. Optimization Algorithm Design

### 4.1. Basic Whale Optimization Algorithm

Humpback whales are excellent hunters in nature. They have a special way of hunting, that is, they produce unique bubbles along the circular or “9”-shaped path to keep close to their prey for hunting. Therefore, the whale optimization algorithm (WOA), which simulates the hunting behavior of humpback whales, is also an algorithm with strong global optimization ability. The hunting behavior of humpback whales mainly includes three stages: surrounding prey, spiral hunting with bubble net, and random search for prey [24].

When humpback whales hunt, they not only surround the prey, but also swim toward it in a spiral motion, and at the same time, they also shrink the encirclement. In addition, humpback whales search randomly for prey during hunting. In the whale optimization algorithm, each individual whale position is the solution of an optimization problem [25]. If the decision variable dimension of the optimization problem is *n*, the whale population size in the basic whale optimization algorithm is NPs, and the update formula of the whale individual position vector $X_{\left(t+1\right)}$ can be expressed as follows:

(27) $$\begin{array}{c} X_{\left(t+1\right)}=\left\{\begin{array}{cc} X_{\left(t\right)}^{*}-A\cdot D & p<P_{s}\;,\;\left|A\right|<1\\ X_{rand}-A\cdot D & p<P_{s}\;,\;\left|A\right|\geq 1\\ X_{\left(t\right)}^{*}+D_{p}e^{bl}\cos\left(2\pi l\right) & p\geq P_{s} \end{array}\right. \end{array}$$

where $D_{p}=\left|X_{\left(t\right)}^{*}-X_{\left(t\right)}\right|=\left\{\left|X_{\left(t\right)}^{*1}-X_{\left(t\right)}^{1}\right|,\ldots ,\left|X_{\left(t\right)}^{*n}-X_{\left(t\right)}^{n}\right|\right\}$ is the *n*-dimensional distance vector between the whale individual position $X_{\left(t\right)}$ and the optimal whale individual position $X_{\left(t\right)}^{*}$; $X_{rand}$ is the *n*-dimensional random whale individual position vector; *p* is the probability of whale behavior selection, $p\in \left[0,1\right]$; $P_{s}$ is the probability of individual whale choosing to try to surround or randomly search for prey; $P_{s}\in \left[0,1\right]$, $1-P_{s}$ is the probability of choosing bubble net spiral hunting behavior for individual whales; *b* is the adjustment coefficient of spiral shape; *l* is the random number in (−1, 1); *A* and *C* are the correlation coefficients.

(28) $$\begin{array}{c} D=\left|CX_{rand}-X_{\left(t\right)}\right| \end{array}$$

where the formula is the *n*-diemensional absolute value vector of the difference between $CX_{rand}$ and the whale individual position $X_{\left(t\right)}$.

The specific calculation formula of correlation coefficients *A* and *C* is shown as follows:

(29) $$\begin{array}{c} \left\{\begin{array}{c} A=2a\times r_{1}-a\\ C=2\times r_{2} \end{array}\right. \end{array}$$

where, $r_{1}$ and $r_{2}$ are random numbers in (0,1); *a* is the convergence factor.

The role of the convergence factor is to balance the exploration and development of population optimization, that is, to balance the relationship between global and local search. In the basic whale optimization algorithm, the convergence factor decreases linearly from 2 to 0 with the number of iterations so that *A* gradually decreases to 0.

The specific convergence factor calculation formula of the basic whale optimization algorithm is shown as follows:

(30) $$\begin{array}{c} a=2-2\times t/T_{\max} \end{array}$$

### 4.2. Non-Linear Decreasing Strategy of Convergence Factor

The important parameters of the optimization algorithm have a certain influence on its optimization performance. If a fixed, the arbitrarily assigned parameter method is used, it is not conducive to achieving better optimization performance [26]. To improve the global search ability of whale optimization algorithm in the early stage of iteration, a larger convergence factor *a* should be selected. On the contrary, in the late stage of iteration, a smaller convergence factor *a* should be selected for enhancing the local search ability.

In this paper, a non-linear decreasing strategy of convergence factor *a* based on exponential decreasing is given. The specific calculation formula of convergence factor *a* is shown as follows:

(31) $$\begin{array}{c} a\left(t_{r}\right)=-0.5+2.5\times e^{{t_{r}}^{\beta_{a}}\times \ln\left(\frac{0.5}{2.5}\right)} \end{array}$$

where $\beta_{a}$ is the optimization factor of non-linear decline of convergence factor *a*.

**Figure 3:**
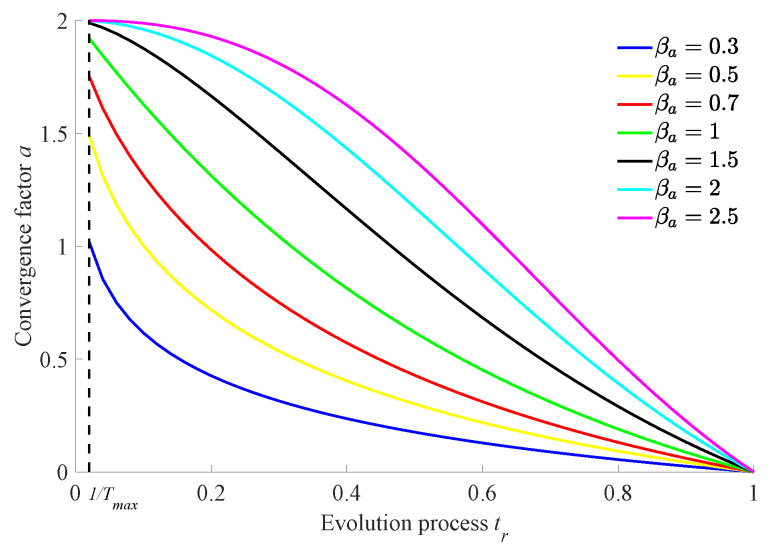
Schematic diagram of the non-linear decreasing function for the convergence factor about the iteration progress.

The specific schematic diagram of the non-linear decreasing function for the convergence factor *a* about the iteration progress $t_{r}$ is shown in Figure 3.

It can be seen from Figure 3 that using the above exponential non-linear decline strategy, the convergence factor *a* decreases non-linearly from 2 to 0 with the iteration progress, and its non-linear decrement trend can be adjusted by choosing the most suitable optimization factor $\beta_{a}$ to improve the global optimization ability of the algorithm as much as possible. This strategy can make the decreasing rate of convergence factor have significant difference in the whole iteration process so as to balance the global and local search ability of the algorithm in real time and to improve the global optimization ability of the algorithm as much as possible.

### 4.3. Solutions of Out-of-Bounds Eliminating Strategy via Combination of Reflection and Refraction

The data range clipping method is a data-processing technique that effectively handles data outside a given reasonable range by adjusting it to fall within the specified bounds.

First, a commonly used data range clipping method is introduced. For the *i*th dimension $x_{j,i}^{k}$ of the *j*th individual in the population at the *k*th iteration, there exists a constraint condition $x_{j,i}^{k}\in \left[a_{i},b_{i}\right]$. If the following relationship holds, the solution $x_{j}^{k}$ is considered an out-of-bounds solution, exceeding the limits in the *i*th dimension.

(32) $$\begin{array}{c} x_{j,i}^{k}<a_{i}\left|\right|x_{j,i}^{k}>b_{i} \end{array}$$

If an n-dimensional out-of-bounds solution $x_{j}^{k}=\left[x_{1}^{k},\ldots ,x_{n}^{k}\right]$ violates constraints in the *i*th dimension (i.e., $x_{j,i}^{k}$ is out of bounds), then for the solution $x_{p,i}^{k}$, if either of the following two conditions holds, $x_{p,i}^{k}$ is referred to as the clipped substitute solution within the valid data range for the *i*th dimension of the out-of-bounds solution $x_{j,i}^{k}$.

If $x_{j,i}^{k}<a_{i}$, the solution $x_{p,i}^{k}$ is defined as

(33) $$\begin{array}{c} x_{p,i}^{k}=\left\{\begin{array}{c} xp_{j,m}^{k}=x_{j,m}^{k}\;\;\;\forall m\neq i\\ xp_{j,i}^{k}=a_{i} \end{array}\right. \end{array}$$

If $x_{j,i}^{k}>b_{i}$, the solution $x_{p,i}^{k}$ is defined as

(34) $$\begin{array}{c} x_{p,i}^{k}=\left\{\begin{array}{c} xp_{j,m}^{k}=x_{j,m}^{k}\;\;\;\forall m\neq i\\ xp_{j,i}^{k}=b_{i} \end{array}\right. \end{array}$$

Adopting this conventional data range clipping strategy for out-of-bounds solutions can effectively address boundary violations in the population. However, it inevitably increases the risk of premature local convergence.

Opposition-Based Learning (OBL) was proposed by Tizhoosh in 2005 [27]. If the *n*-dimensional solution out of bounds $x_{j}^{k}=\left[x_{1}^{k},\ldots ,x_{n}^{k}\right]$ is out of bounds in the *i*th dimension, for the solution $xo_{j}^{k}$, if the following relation is true, then solution $xo_{j}^{k}$ is called the reverse solution for the solution out of bounds $x_{j}^{k}$ in the *i*th dimension.

(35) $$\begin{array}{c} xo_{j}^{k}=\left\{\begin{array}{c} xo_{j,m}^{k}=x_{j,m}^{k}\;\;\;\forall m\neq i\\ xo_{j,i}^{k}=a_{i}+\beta \left(b_{i}-xo_{j,i}^{k}\right) \end{array}\right. \end{array}$$

where β is the attenuation coefficient, $\beta \in \left(0,1\right)$.

It can not only effectively deal with the out-of-bounds problem of the solution via introducing the reverse solutions, but also expand the search range of the algorithm to a certain extent. But as the number of iterations increases, there occurs a phenomenon that the reverse solutions fall into the local optimal.

To address this deficiency, the following solution of out-of-bounds elimination strategy via combining reflection and refraction is adopted.

Reflection and refraction are two fundamental optical phenomena. When light travels from one transparent medium to another, part of it is reflected at the interface, while the remaining portion passes into the second medium, changing direction due to refraction. Reflection occurs when light bounces off the surface of the new medium, whereas refraction happens when light alters its path as it enters a medium with a different refractive index. If the n-dimensional solution out of bounds $x_{j}^{k}=\left[x_{1}^{k},\ldots ,x_{n}^{k}\right]$ is out of bounds in the *i*th dimension, it is assumed that the *i*th dimension is the interface, and the incident height $I_{H}$ is $\min\left(\left|x_{j,i}^{k}-a_{i}\right|,\left|x_{j,i}^{k}-b_{i}\right|\right)$, where $\left|x_{j,i}^{k}\right|$ is the absolute value of $x_{j,i}^{k}$. For solution $xr_{j}^{k}$, if the following relation is true, then solution $xr_{j}^{k}$ is called the refractive solution for the solution out of bounds $x_{j}^{k}$ in the *i*th dimension.

(36) $$\begin{array}{c} xr_{j}^{k}=\left\{\begin{array}{c} xr_{j,m}^{k}=x_{j,m}^{k}\;\;\;\forall m\neq i\\ xr_{j,i}^{k}=\frac{a_{i}+b_{i}}{2}+\frac{\left(\frac{a_{i}+b_{i}}{2}-I_{H}\right)}{f\cdot n_{r}} \end{array}\right. \end{array}$$

where *f* is the projection ratio, f>1 and $n_{r}$ is the refractive index. In this study, the refractive index used is defined as the reciprocal of the optical refractive index. Specifically, the optical refractive index of the selected metal is set to 3 and the optical refractive index of the selected polluted air is set to 1.05. $n_{r}$∈ (0.33, 0.95).

Assuming that the maximum refraction number is $f_{\max}$, the refraction number $f_{n}$ is calculated as follows:

(37) $$\begin{array}{c} f_{n}=\min\left(\left\lceil \frac{f_{\max}I_{H}}{\left|b_{i}-a_{i}\right|}\right\rceil ,f_{\max}\right) \end{array}$$

where ⌈⌉ is the carry up integer operator.

From this, both refraction and reflection are further learning (deep mining) behaviors based on preserving existing optimization results. It is also known that the refractive solution $xr_{j}^{k}$ is not necessarily out of bounds, but the inverse solution $xo_{j}^{k}$ must not be out of bounds. Hence, through $f_{n}$-timesrefraction learnings and one-time reflection (reverse) learning, the optimal solution that is not out of bounds can be found. By adopting the solution of out-of-bounds elimination strategy that combines reflection and refraction as described above, the global exploration strength of the algorithm is improved: through reverse learning, a reverse solution is obtained, if the reverse solution is still far from the optimal solution, multiple refractive solutions (not exceeding the maximum refraction number $f_{\max}$) is obtained through repeated refraction operations. The maximum refraction number $f_{\max}$, projection ratio *f*, refractive index $n_{r}$, and attenuation coefficient β can all be adjusted, which enhances the adaptability of the solution of the out-of-bounds elimination mechanism.

In this study, the maximum refraction number $f_{\max}$ is set to a fixed value, while the projection ratio *f*, refractive index $n_{r}$, and attenuation coefficient β are all random numbers within a certain range.

### 4.4. Genetic Evolution Mechanism with Variable Probability

The WOA is based on the current optimal position for optimization so that it has a certain direction guidance, which is beneficial for accelerating convergence and global optimization. But this makes the evolution of whale populations overly dependent on optimal positions so as to result in a loss of diversity. Thus, it is not conducive to global convergence. The genetic evolution mechanism can continuously generate a large number of new solutions with significant differences through selection, crossover, and mutation so as to better maintain population diversity; thus, it is conducive to global convergence [28]. Based on the above reasons, the multi-objective whale optimization algorithm designed in this study adopts an evolutionary method that combines whale population updating with genetic evolution mechanism and adopts the comprehensive evaluation strategy for target speed curve optimization of urban rail train based on APD so as to enhance the rationality and practicality of the evaluation. If a fixed and blindly random mutation probability calculation method is used, it is not conducive to obtaining better global optimization effect. Based on the quality ranking value of each whale individual in the whale population, their corresponding probabilities of selection, mutation, and crossover are assigned.

(38) $$\left\{\begin{array}{cc} p_{s_{i}} & =p_{s\;\min}+\left(\frac{i-1}{NPs-1}\right)\times \left(p_{s\;\max}-p_{s\;\min}\right)\\ p_{c_{i}} & =p_{c\;\min}+\left(\frac{i-1}{NPs-1}\right)\times \left(p_{c\;\max}-p_{c\;\min}\right)\\ p_{m_{i}} & =p_{m\;\min}+\left(\frac{i-1}{NPs-1}\right)\times \left(p_{m\;\max}-p_{m\;\min}\right) \end{array}\right.$$

where $p_{s_{i}}$, $p_{c_{i}}$, and $p_{m_{i}}$ are, respectively, the probabilities of selection, crossover, and mutation of the individual whale ranked *i*th in terms of quality; $p_{s\;\min}$, $p_{c\;\min}$, $p_{m\;\min}$, and $p_{s\;\max}$, $p_{c\;\max}$, $p_{m\;\max}$ are the minimum and maximum probabilities of individual whale selection, crossover, and mutation, respectively; NPs refers to the total count of whales in a given population.

### 4.5. Elite Maintenance Mechanism Based on Fusion Distance and Crowding Degree Distance

To better solve the complex practical multi-objective optimization problems, the elite preservation mechanism serves as a pivotal component that systematically maintains high-quality solutions while ensuring balanced convergence and diversity within the optimization framework. Usually, in the process of each iteration update, the screened non-dominated solutions of the population are extended into the elite concentration to better preserve the existing optimization results. To prevent the rapid increase in the number of elites in the elite set from affecting the computational efficiency of the algorithm, it is necessary to maintain the size of the elite set. Here, $N_{E}$ (Number of Elites) represents the current count of non-dominated solutions stored in the elite set, while $N_{ES}$ (Number of Elite Set) denotes the preset maximum capacity, that is, $N_{E}\leq N_{ES}$.

At present, the elite selection mechanism based on Pareto domination and the elite set maintenance mechanism based on crowding degree distance are widely adopted in multi-objective optimization algorithms. However, the crowding degree distance can only maintain the dispersion of each object in the solution object space of the elite set, but it cannot effectively suppress the defect of the aggregation phenomenon in the solution space of the elite set. A type of algorithms that rely on learning the optimal individual for population evolution, such as WOA, moth optimization algorithm, and particle swarm algorithm, is prone to clustering towards the optimal individual in the late stages of its evolution process, which is not conducive to the algorithm jumping out of local optima [29]. Traditional multi-objective optimization algorithms use calculating the Euclidean distance between solutions in each solution space to delete denser solutions. However, the calculation of Euclidean distance depends on the dimensionality between variables, and Euclidean distance is calculated as the straight-line distance between samples, which cannot measure the correlation between variables [30]. Based on this, Liu Gang et al. proposed a fusion distance that fuses the Mahalanobis distance and the Euclidean distance, and its specific calculation formula is shown in the following equation [31]:

(39) $$\begin{array}{c} \left\{\begin{array}{c} d_{Mix}=\omega \times MD\left(X,Y\right)+\left(1-\omega \right)\times ED\left(X,Y\right)\\ C_{Y}=\left[\begin{array}{c} \begin{array}{c} \rho_{Y_{1}Y_{1}} \end{array}\rho_{Y_{1}Y_{2}}\ldots \rho_{Y_{1}Y_{n}}\\ \rho_{Y_{2}Y_{1}}\rho_{Y_{2}Y_{2}}\ldots \rho_{Y_{2}Y_{n}}\\ \vdots \vdots \ddots \vdots \\ \rho_{Y_{n}Y_{1}}\rho_{Y_{n}Y_{2}}\ldots \rho_{Y_{n}Y_{n}} \end{array}\right]\\ \omega =\sqrt{1-\left|C_{Y}\right|} \end{array}\right. \end{array}$$

where *Y* represents the sample set and *X* represents the test sample. In the calculation of the average fusion distance, the sample set Y corresponds to the elite set $\Omega_{E}$, and the test sample X represents an individual elite solution within $\Omega_{E}$; $d_{Mix}$ represents the fusion distance; MD represents the Mahalanobis distance; $MD{\left(X,Y\right)}^{2}=Z^{\prime }C_{Y}Z$, *Z* represents the standardized sample of the test sample *X*; ED represents the Euclidean distance; $ED{\left(X,Y\right)}^{2}=Z^{\prime }Z$, $C_{Y}$ represents the correlation coefficient matrix of sample set *Y*; *n* represents the number of samples in sample set *Y*; and $Y_{i}\left(i=1,\ldots ,n\right)$ represents the samples in sample set *Y*. $\rho_{XY}$ represents the correlation coefficient between sample *X* and sample *Y*, $\rho_{XY}=\frac{Cov(X,Y)}{\sqrt{D(X)}\sqrt{D(Y)}}$, where Cov(X,Y)=E(XY)−E(X)E(Y), is the covariance of sample *X* and sample *Y*, and D(X) and E(X) are the variance and expectation of sample *X*, respectively. Since Mahalanobis distance takes into account the correlation between variables, ω with relevant information weight is used for fusion, while Euclidean distance is fused with 1−ω.

Taking into account both the target dispersion of the elite set in the solution target space and the elite clustering in the solution space, this paper uses fusion distance and crowding degree distance to maintain the elite set. Specifically, when the size of the elite set exceeds the predefined limit, individuals are ranked based on the average of their fusion distance and crowding degree distance. A sufficient number of low-ranking individuals, those with larger average fusion and crowding degree distances, are eliminated from the elite set. If multiple individuals share the same average ranking, they are further reordered according to their crowding degree distance to prioritize solutions with better distribution. To illustrate the elite set maintenance mechanism presented in this paper, the following assumptions are made. Assuming that the elite set waiting for maintenance contains six elites, the relevant distance ranking of each elite in elite set is shown in Table 2.

(40) $$\begin{array}{c} D_{i}=\sum_{m=1}^{M}\frac{f_{i+1}^{m}-f_{i-1}^{m}}{f_{\max}^{m}-f_{\min}^{m}} \end{array}$$

where $f_{i}^{m}$ represents the value of the *i*th individual on the *m*th objective function; $f_{\max}^{m}$ and $f_{\min}^{m}$ represent the maximum and minimum values on the *m*th objective function; $f_{i+1}^{m}$ and $f_{i-1}^{m}$ represent the objective function values of the neighboring individuals relative to the current individual, after sorting by the *m*th objective.

In this study, all ranking procedures are performed using the bubble sort algorithm to ensure consistency and stability. To comprehensively evaluate solution quality, the Comprehensive distance ranking is constructed. This ranking is derived from the intermediate metric the Mean distance ranking, which integrates two perspectives: the Crowding distance ranking and the Fusion distance ranking. The Mean distance ranking is calculated as their arithmetic average:

(41) $$\begin{array}{c} d_{M}=\frac{d_{C}+d_{F}}{2} \end{array}$$

where $d_{M}$ represents the value of Mean distance ranking; $d_{C}$ represents the value of Crowding distance ranking; $d_{F}$ represents the value of Fusion distance ranking.

In Table 2, distance comprehensive ranking is determined by the mean value for fusion distance and crowding degree distance ranking. Since Elite 1 and Elite 4 are tied for 4th place, Elite 7 is ranked 6th. If the predetermined size $N_{ES}$ of the elite set is 6, only Elite 2 with the distance comprehensive ranking first is screened out. If the predetermined size $N_{ES}$ of the elite set is 5, then Elite 5, which is the distance comprehensive ranking second, also needs to be screened out.

### 4.6. Design of the IWOA-APD

In the WOA, the optimal whale individual position has a significant impact on the evolution of whale populations. Although this evolutionary approach is beneficial for improving convergence speed, it is not conducive to maintaining population diversity, so the population is prone to fall into local optimal in the late iteration. Therefore, in the iterative calculation process, the variable probability genetic evolution mechanism is introduced to better maintain population diversity so as to improve the global optimization performance of the whale optimization improvement algorithm. The evaluation indexes for the quality of solutions are also particularly important. This paper adopts the angle penalized distance APD as the evaluation index and proposes a comprehensive evaluation strategy based on decision-makers’ preferences. For the adaptive assignment of important parameter convergence factors, maintenance of elite set, and elimination of the solution out of bounds, this study adopts a non-linear decreasing strategy based on exponential form, evaluation criteria combining fusion distance and crowding distance, and solution of out-of-bounds elimination strategy via combining reflection and refraction, respectively.

**Figure 4:**
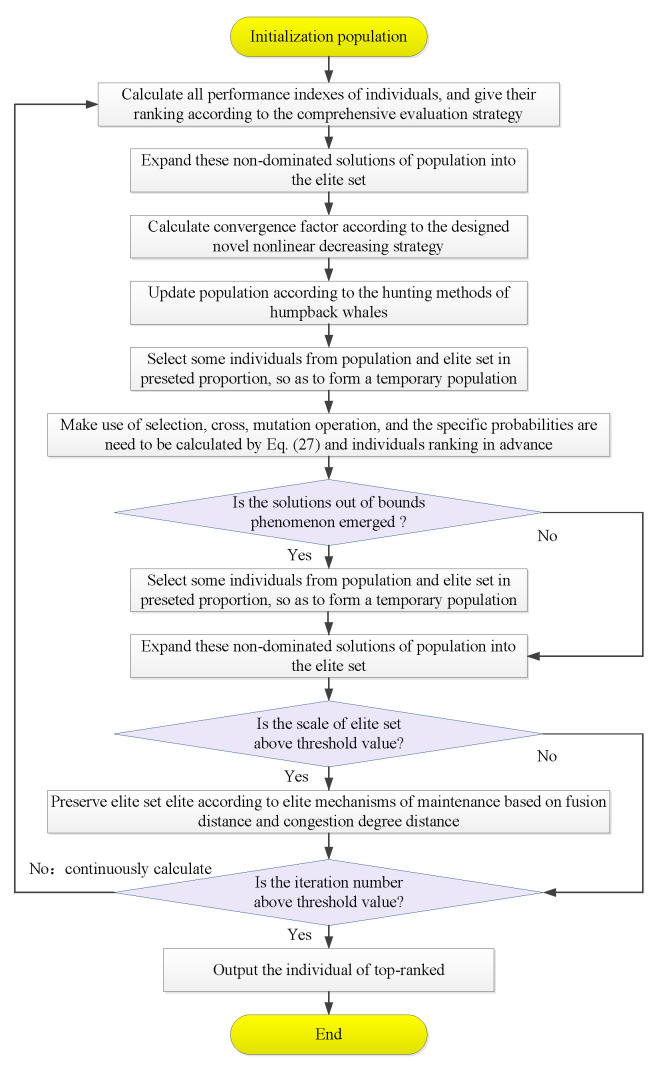
Calculation process diagram of improved whale optimization algorithm based on APD.

The specific calculation process diagram of the improved whale optimization algorithm based on APD is shown in Figure 4.

In Figure 4, if the selection or crossover operator is performed, the whale individuals in the temporary population with the minimum difference from the original whale individuals (if the 2-norm distance between the two solutions is greater than 0, then they have a certain difference) are used as the selected replacement or crossed whale individuals.

## 5. Simulation and Experimental Verification

### 5.1. Main Parameter Settings of IWOA-APD

In this study, empirical parameters—including the random prey search probability, spiral adjustment coefficient, and optimization factor—were initialized using validated values from the literature [32] and subsequently fine-tuned to meet the specific requirements of the proposed optimization framework. Therefore, the probability $P_{s}$ of individual whale choosing to try to surround or randomly search for prey was 0.55, the adjustment coefficient of spiral shape *b* was 1, and the optimization factor $\beta_{a}$ of non-linear decline of convergence factor *a* was 1.75.

Then, based on the initially defined optimization targets, which were an IGD value of $1\times 10^{-3}$ and a computation time of 140 s, a two-level factorial experiment consisting of 12 trials was conducted. By balancing the trade-off between computational resource limitations, which constrain the acceptable population size, and the demand for higher optimization performance, which favors larger populations, a well-balanced parameter setting strategy was determined. The following parameter values were adopted: the whale population size NPs was 50, the maximum number of iterations $T_{\max}$ was 100, the predetermined size $N_{ES}$ of the elite set was 120, the temporary population size Nt was 80 and its elite individual proportion was 60%, and the maximum refraction number $f_{\max}$ was 5. Under this configuration, the proposed IWOA-APD algorithm was applied to the ZDT1 benchmark problem. The final optimization outcome achieved an IGD value of $8.4\times 10^{-4}$ and a total computation time of 108 s, thereby meeting the predefined optimization criteria and demonstrating strong performance.

**Figure 5:**
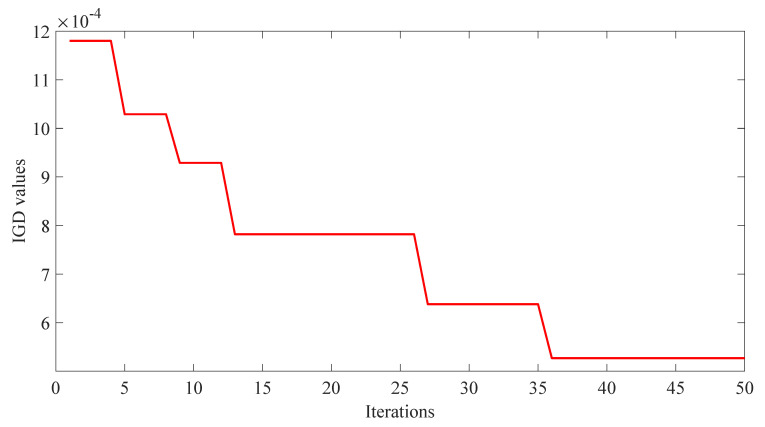
The IGD iteration curves of the complex tuning parameters by using the genetic algorithm.

Finally, based on empirical parameters and preliminary tuning, a convergence optimization experiment was then conducted using a genetic algorithm to fine-tune seven groups of complex parameters. The corresponding IGD convergence curves were obtained to evaluate the optimization performance. Detailed results regarding the integrated optimization parameters obtained in the ZDT1 scenario are presented in Table 3. The IGD iteration curves of the complex tuning parameters by using the genetic algorithm are shown in Figure 5.

Since the experimental benchmark platform (including ZDT1, ZDT2, DTLZ1, and DTLZ2 problems) shares similar multi-objective optimization structures and objective characteristics, there is no substantial difference in algorithm behavior across these scenarios. Therefore, the key parameters of the IWOA-APD algorithm obtained through optimization under the ZDT1 scenario are also applicable to the other three scenarios and similar multi-objective optimization problems. In this paper, the optimization parameter settings derived from the ZDT1 benchmark were consistently applied in the ZDT2, DTLZ1, DTLZ2 benchmark functions and target speed curve optimization scenarios.

### 5.2. Selection of Comparative Verification Algorithms

In the context of urban rail train speed profile optimization, which is subject to stringent industry regulations and high operational safety requirements, it is essential to adopt optimization algorithms that are not only highly reliable but also validated through practical applications. Therefore, to verify the effectiveness of the proposed improved whale optimization algorithm with adaptive parameter distribution (IWOA-APD), two commonly used and well-established algorithms were selected as benchmarks: the decomposition-based Multi-objective Particle Swarm Optimization (dMOPSO) [33] and the multi-objective whale optimization algorithm (MOWOA) [34].

The dMOPSO algorithm decomposes a multi-objective problem into a series of scalar sub-problems, each of which is optimized using a particle swarm optimization strategy. This approach maintains a good balance between convergence and diversity. MOWOA, as a representative extension of the (WOA) for multi-objective problems, integrates techniques such as crowding distance calculation and elite solution preservation to effectively manage trade-offs between conflicting objectives. Its application effectiveness has also been demonstrated in various engineering scenarios.

Compared with these algorithms, the proposed IWOA-APD introduces an adaptive parameter distribution mechanism to enhance population diversity and improve the uniformity of the Pareto front distribution while preserving the core exploitation and exploration capabilities of the original WOA. Therefore, the selection of dMOPSO and MOWOA as comparison algorithms ensures a fair and comprehensive evaluation of the proposed method, balancing practicality and effectiveness.

### 5.3. Simulation Results and Analysis Based on Benchmark Functions

**Figure 6:**
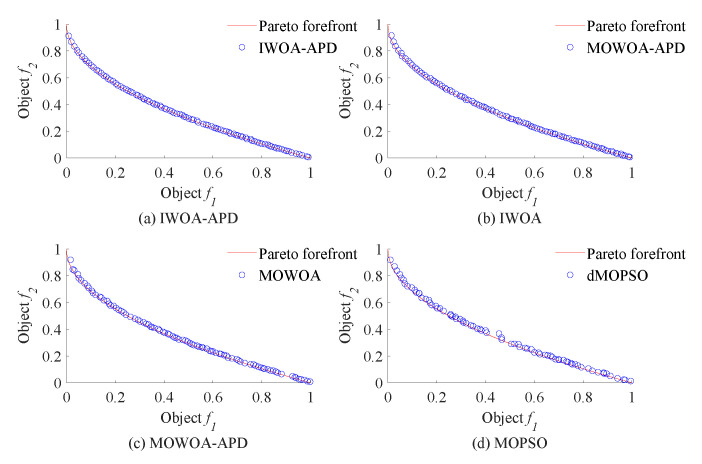
Optimization simulation results using ZDT1 benchmark function.

**Figure 7:**
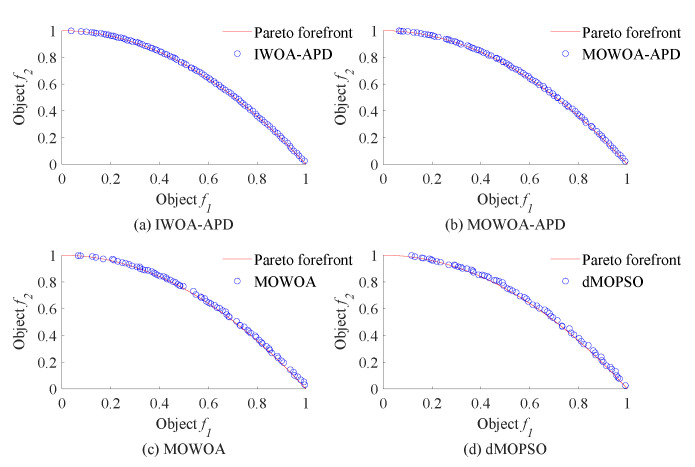
Optimization simulation results using ZDT2 benchmark function.

**Figure 8:**
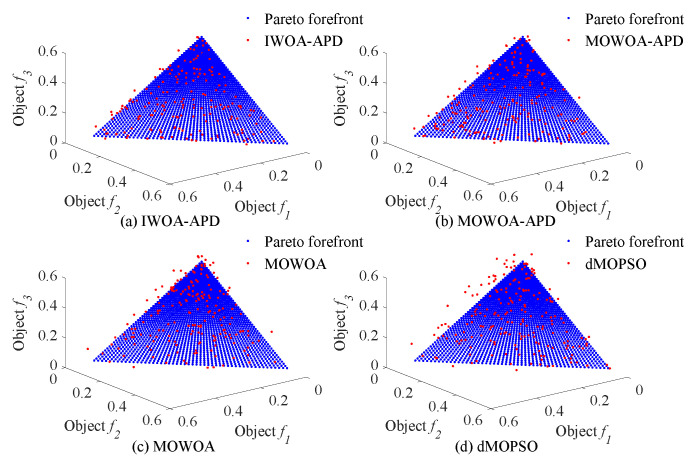
Optimization simulation results using DTLZ1 benchmark function.

**Figure 9:**
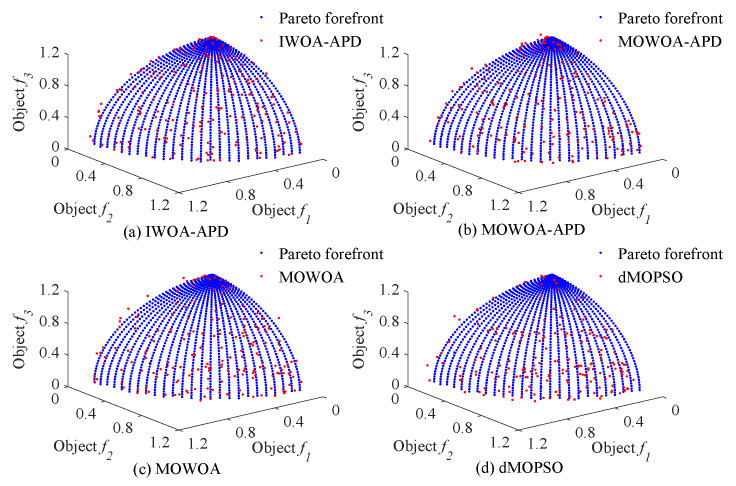
Optimization simulation results using DTLZ2 benchmark function.

**Figure 10:**
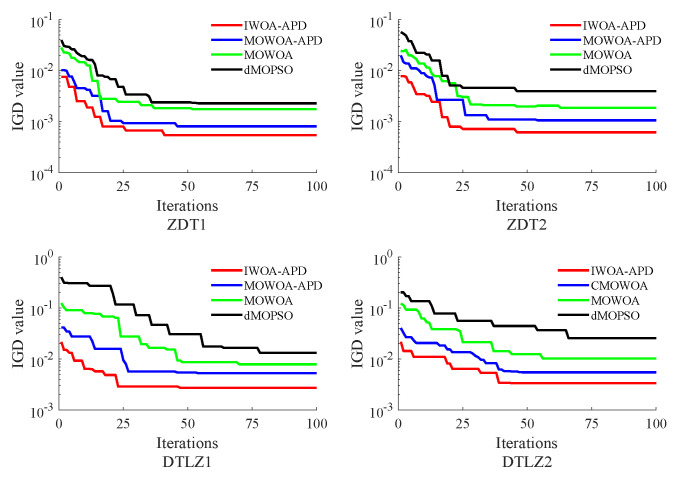
IGD value iterative convergence curves of each optimization algorithm.

In this study, the benchmark functions simulation verification platform based on MATLAB was built, and its main configurations were as follows: the version of MATLAB was 2016b, and the computer performance was configured as CPU Core i7-7770k. In this study, two benchmark functions with different properties in ZDT [33] series of double objective benchmark functions and DTLZ [35] series of two objectives benchmark functions were used as multi-objective optimization objects. To verify the effectiveness of the IWOA-APD, the ZDT1, ZDT2, DTLZ1, and DTLZ2 benchmark functions were optimized by IWOA-APD, MOWOA-APD, MOWOA, and dMOPSO under the same conditions based on the above simulation verification platform, and the optimization results were compared and analyzed. The specific optimization simulation results using four benchmark functions are shown in Figure 6, Figure 7, Figure 8 and Figure 9. The specific IGD value iterative convergence curves of each optimization algorithm are shown in Figure 10. The IGD values obtained by each multi-objective optimization algorithm and their calculation time taken are shown in Table 4 and Table 5.

In Figure 6 and Figure 7, the real Pareto frontier is represented by a solid red line, and the elite individuals found by the optimization algorithm are represented by a blue hollow circle. In Figure 8 and Figure 9, the real Pareto solution is represented by a blue solid circle, and the elite individuals found by the optimization algorithm are represented by a red solid circle.

It can be seen from Table 4 and Table 5 that IWOA-APD has significantly better global optimization performance. Compared with the multi-objective optimization algorithms MOWOA-APD, MOWOA, and dMOPSO used for comparison and verification, IWOA-APD can obtain significantly better optimization performance indexes, and the calculation time is shorter. It can be seen from Figure 6, Figure 7, Figure 8 and Figure 9 that for ZDT series double-objective benchmark functions and DTLZ series three-objective benchmark functions, compared with the multi-objective optimization algorithms MOWOA-APD, MOWOA, and dMOPSO used for comparison and verification, IWOA-APD can obtain the optimization frontier closer to its real Pareto frontier, the dispersion of the optimization front is better, and the distribution of each optimization solution is more uniform. It can be seen from Figure 10, compared with the multi-objective optimization algorithms MOWOA-APD, MOWOA, and dMOPSO used for comparison and verification, that IWOA-APD not only obtains significantly better optimization performance index IGD value, but also achieves significantly faster convergence speed.

In this study, the evaluation of the IGD metric relies on prior knowledge of the true Pareto front. Although the theoretical validity is ensured by using a uniformly sampled point set $P^{*}$, the unknown nature of the true Pareto front in practical applications may limit the general applicability of this metric. Future work will explore alternative evaluation methods that do not depend on the true Pareto front.

### 5.4. Introduction for Target Speed Curve Optimization Scenarios

In this study, the target speed curve optimization scenarios selected as the research objects were as follows: from Lvshun New Port Station to Tieshan Town Station of Dalian urban rail transit line No.12 and from Jiuli Station to 19th Bureau Station of the second-phase project of Dalian urban rail transit line No.13. The operating lengths of target speed curve optimization scenarios from Lvshun New Port Station to Tieshan Town Station and from Jiuli Station to 19th Bureau Station are 2.94 km and 2.74 km, respectively, and there are 2 speed limit sections and 3 long steep slope sections in both scenarios. Dalian urban rail transit line No.12 starts from Hekou Station of Dalian High-tech Park to Lvshun New Port Station of Dalian Lvshun Economic and Technological Development Zone, with a total length of 40 km and 8 stations. The second-phase project of Dalian urban rail transit line No.13 starts from Dalian North Station in Ganjingzi District of Dalian to Jiuli in Jinzhou District of Dalian, with a total length of 22 km and 8 stations. To simplify the description, the target speed curve optimization scenario of urban rail trains from Lvshun New Port Station to Tieshan Town Station of Dalian urban rail transit line No.12 was recorded as Optimization Scenario 1, and the target speed curve optimization scenario from Jiuli Station to 19th Bureau Station of the second-phase project of Dalian urban rail transit line No.13 was recorded as Optimization Scenario 2.

**Figure 11:**
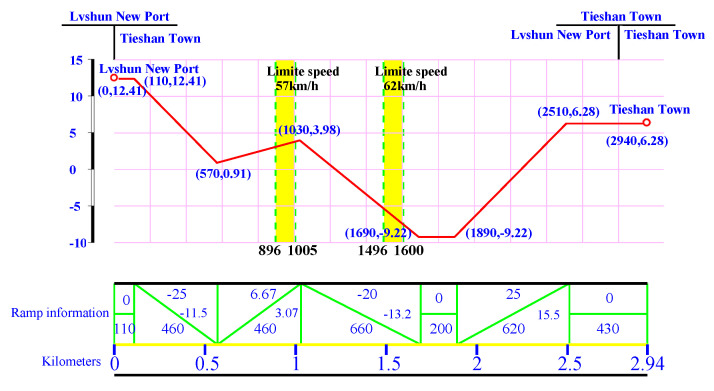
Ramp path trajectory for Optimization Scenario 1.

**Figure 12:**
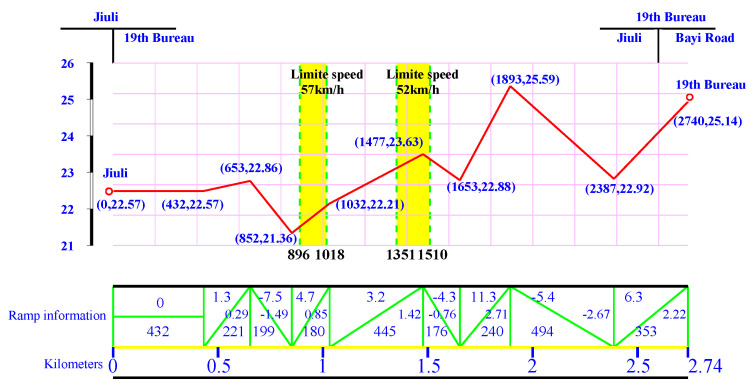
Ramp path trajectory for Optimization Scenario 2.

According to the urban rail train traction calculation standards in China, the vehicle weight (t), maximum speed limit (km/h), and inter-station distance (m) are determined based on actual measurements. Before delivery, urban rail trains undergo professional weighing to determine their weight, and the maximum speed limit is defined accordingly. Prior to trial operation, the inter-station distances are measured using torque-based distance measurement techniques. These measured values and speed limits are not required to retain decimal precision. The maximum allowable stopping error and punctuality error are ±0.2 m and ±0.3 s, respectively. The expected travel time (s) is reasonably determined by the urban rail operator based on real operational conditions. The main parameters of the section from Optimization Scenario 1 and Optimization Scenario 2 are shown in Table 6 and Table 7, respectively. The corresponding train route trajectories are illustrated in Figure 11 and Figure 12.

Figure 11 and Figure 12 illustrate the elevation changes and ramp information during the train’s journey. In Figure 11, the x-axis represents cumulative travel distance (km), and the y-axis represents relative elevation (m). For example, the elevation at the “Lvshun New Port” station is 12.41 m, and at the “Tieshan Town” station, it is 6.28 m. The red line shows the elevation changes at each mileage point, where (a, b) represents the mileage (a, in meters) and relative elevation (b, in meters). Yellow vertical bars indicate speed limit sections, with bold text displaying the speed limits (km/h). The green line represents the ramp information, and the blue numbers show the slope, elevation change, and ramp length (in meters). Slope information is crucial for traction control and energy optimization.

### 5.5. HIL Experiment System Overall Design and Platform Architecture

The (HIL) test system for target speed curve optimization and tracking control is composed of three subsystems: the dSPACE modeling and simulation system, the signal processing system, and the real ATO core function verification system [36]. The dSPACE modeling and simulation system is composed of “HIL dSPACE emulator” and “data recorder”: the “data recorder” stores the big data of the actual historical ATO process; hence, it is the foundation for the real modeling; “HIL dSPACE emulator” adopts DS series processor board and uses the RTI module to realize seamless connection with Matlab/Simulink. It is based on real historical data, real-time signal acquisition, and a large number of electrical, network, and dynamic principles related to the ATO for modeling [37]. The signal processing system adjusts the electrical and network consistency of the interaction signals between the signal of the tested system and the simulation computer board. It contains a large number of “conditioning circuits” and “signal processing units”, and the main communication protocol is Multifunction Vehicle Bus MVB. The real ATO core verification system consists of the real “optimizer” of the“upper optimization loop” and the real “tracking controller” of the “lower control loop”, both of which contain processor chips that write optimization and control algorithms. Its model is “TMS320F28335” [38].

**Figure 13:**
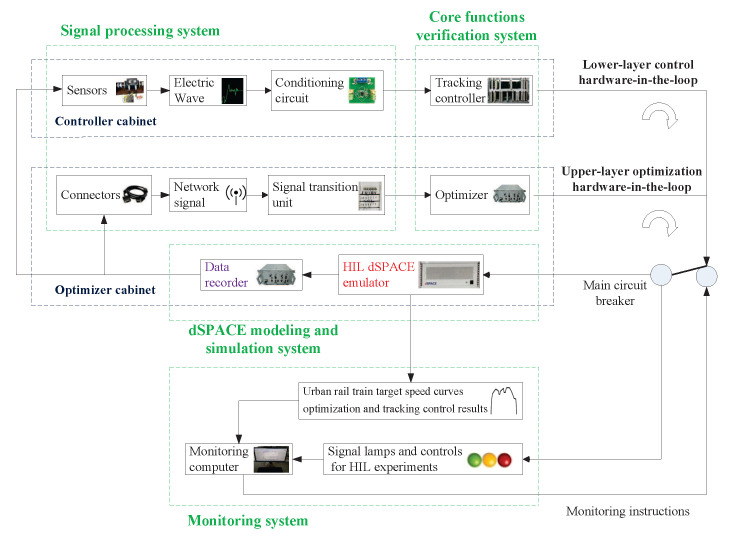
Hardware system overall design diagram for the HIL experiment on target speed curve optimization and tracking control.

**Figure 14:**
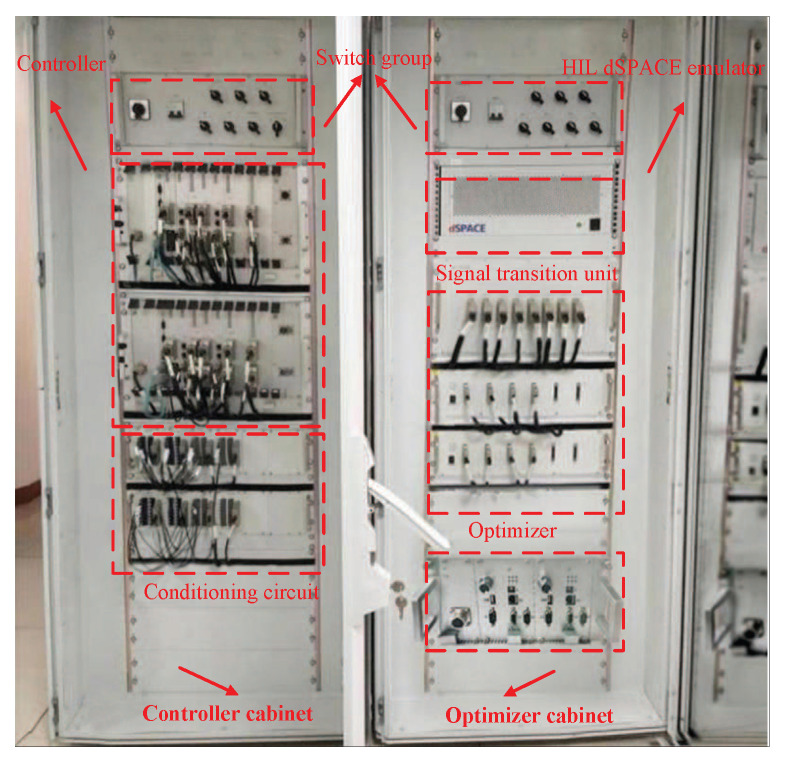
Platform physical photo for the HIL experiment on target speed curve optimization and tracking control.

To better monitor and manage the real-time HIL test process for target speed curve optimization and tracking control of the urban rail train, control desk software is installed on the monitoring upper computer. Control desk software is a supporting testing software for the dSPACE real-time test platform, which has the advantages of convenient hardware management, visual monitoring of real-time test process, and automatic implementation of process test. During the HIL experiment, control desk visually displays the real-time HIL experiment results for target speed curve optimization and tracking control obtained by HIL dSPACE emulator, and the real-time state of the HIL test system is obtained by the state indicator and fault processing device, thus providing the basis for the test and monitoring personnel to impose monitoring instructions. The system overall design and the platform physical photo of the specific target speed curve optimization and tracking control HIL experiment are shown in Figure 13 and Figure 14.

It can be seen from Figure 13 and Figure 14 that the HIL dSPACE emulator cabinet contains the dSPACE modeling system and the hardware equipment required by the upper-layer optimization HIL link, also known as the optimizer cabinet; the controller cabinet contains the hardware devices required by the lower-layer control HIL link. In Figure 14, the controller cabinet and optimizer cabinet are embedded with multiple sub-chassis to achieve different functions, and each sub-chassis encapsulates are fixed the corresponding required pluggable boards.

### 5.6. HIL Test Results and Analysis for Target Speed Curve Optimization and Tracking Control

**Figure 15:**
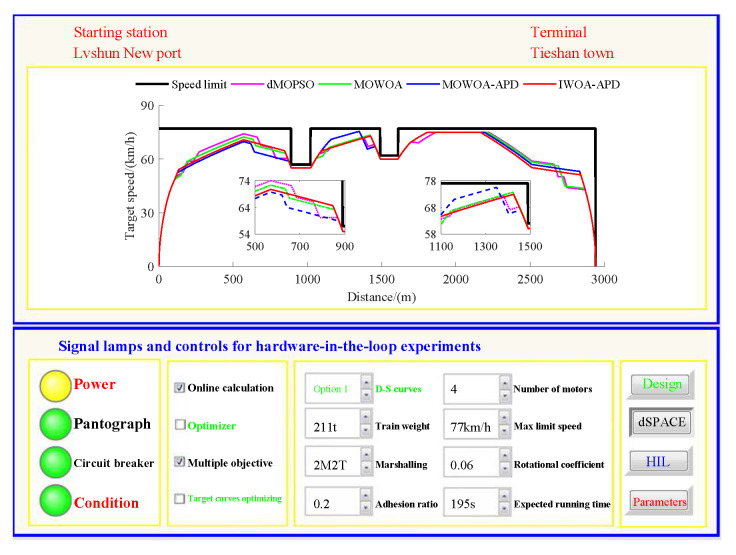
Target speed–distance curves obtained by each optimization algorithm in Optimization Scenario 1.

**Figure 16:**
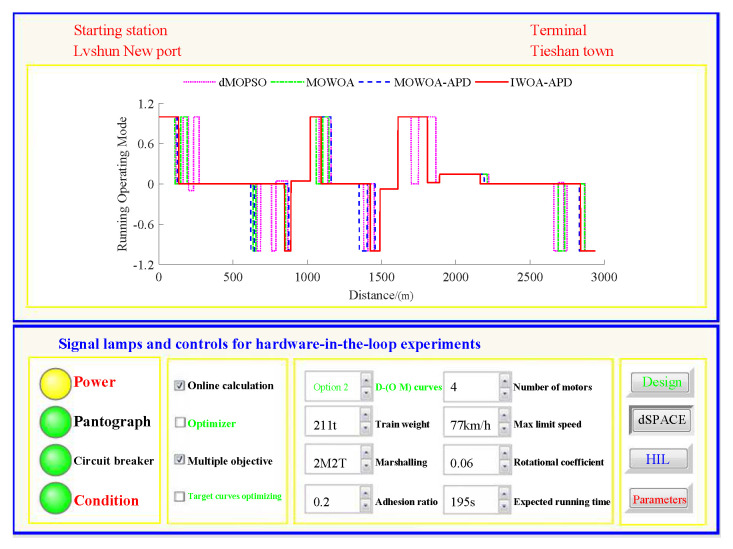
Ideal (running operating mode) curves obtained by each optimization algorithm in Optimization Scenario 1.

**Figure 17:**
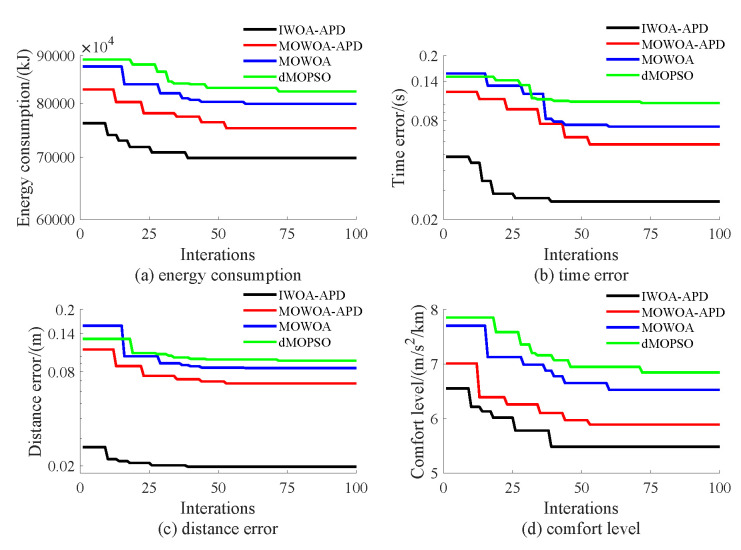
Iteration optimization curves obtained by each optimization algorithm in Optimization Scenario 1.

**Figure 18:**
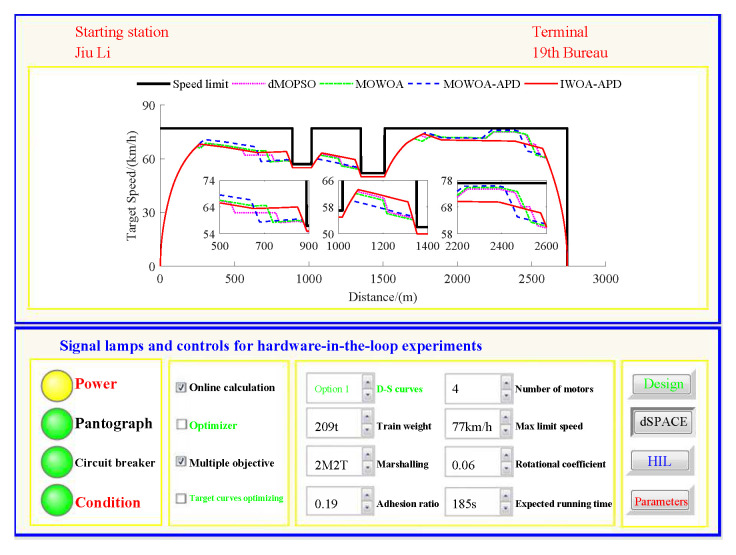
Target speed-distance curves obtained by each optimization algorithm in Optimization Scenario 2.

**Figure 19:**
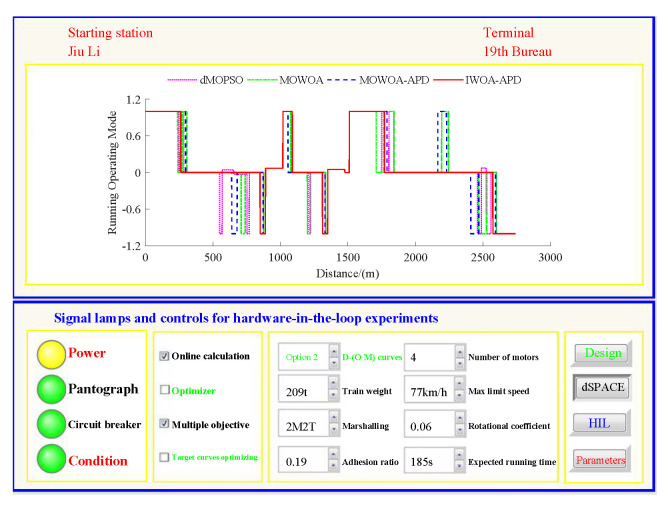
Ideal (running operating mode) curves obtained by each optimization algorithm in Optimization Scenario 2.

**Figure 20:**
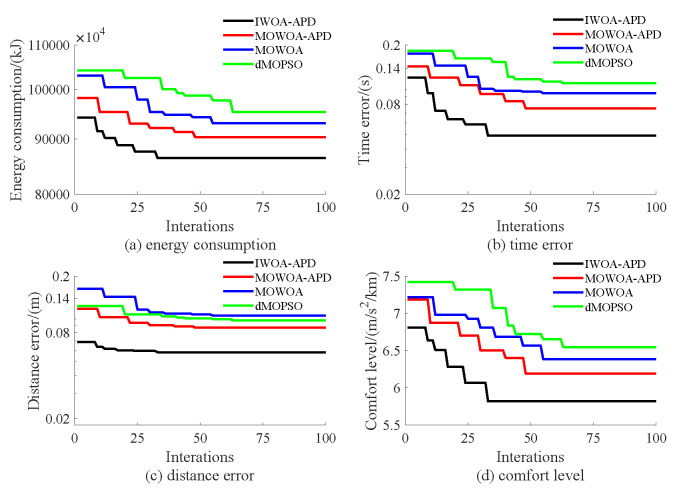
Iteration optimization curves obtained by each optimization algorithm in Optimization Scenario 2.

Based on the target speed curve optimization scenarios from Lvshun New Port Station to Tieshan Town Station of Dalian urban rail transit line No.12 and from Jiuli Station to 19th Bureau Station of the second-phase project of Dalian urban rail transit line No.13 (Optimization Scenario 1 and Optimization Scenario 2), under the above HIL experimental platform for target speed curve optimization and tracking control, IWOA-APD, MOWOA-APD, MOWOA, and dMOPSO were applied to optimize the train target speed curves. The specific HIL test results for target speed curve optimization (including target speed-distance curves, ideal (running operating mode)-distance curves, iteration optimization curves, performance index optimization results, and calculation time by each optimization algorithm) are shown in Figure 15, Figure 16, Figure 17, Figure 18, Figure 19 and Figure 20 and Table 8, Table 9 and Table 10, respectively.

In Figure 15, Figure 16, Figure 17, Figure 18, Figure 19 and Figure 20, the power was turned on, the virtual pantograph module was in a normal state, the main circuit breaker was closed normally, and there was no abnormal situation in HIL test environment for the target speed curve optimization. According to Table 8, Table 9 and Table 10, it can be seen that under the same HIL experimental environment of train target speed curve optimization, IWOA-APD has significantly better global optimization performance; compared with the multi-objective optimization algorithms MOWOA-APD, MOWOA, and dMOPSO used for comparative verification, it obtained significantly better performance index optimization results (energy saving, punctuality, comfort level, and parking accuracy were all improved to a considerable extent), and IWOA-APD requires less computation time on the premise of obtaining significantly better performance index optimization results. From Figure 15 and Figure 18, it can be seen that compared to the multi-objective optimization algorithms MOWOA-APD, MOWOA, and dMOPSO used for comparative verification, IWOA-APD can obtain a more ideal target speed curve so as to enable urban rail train maintain an appropriate speed more smoothly. In Figure 16 and Figure 19, a comparsion can be seen to the multi-objective optimization algorithms MOWOA-APD, MOWOA, and dMOPSO used for comparative verification.

The ideal operating mode sequence obtained by IWOA-APD optimization is more concise and can avoid unnecessary operations to the greatest extent, thus ensuring that its corresponding target speed curve is the smoothest to the greatest extent. From Figure 17 and Figure 20, it can be seen that compared to the multi-objective optimization algorithms MOWOA-APD, MOWOA, and dMOPSO used for comparative verification, IWOA-APD not only has better global convergence performance, but also has higher computational efficiency and faster convergence speed.

**Figure 21:**
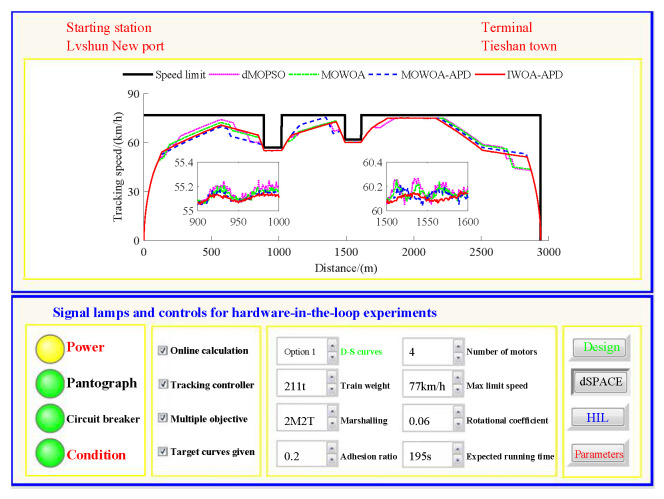
Tracking speed–distance curves obtained by each optimization algorithm in Optimization Scenario 1.

**Figure 22:**
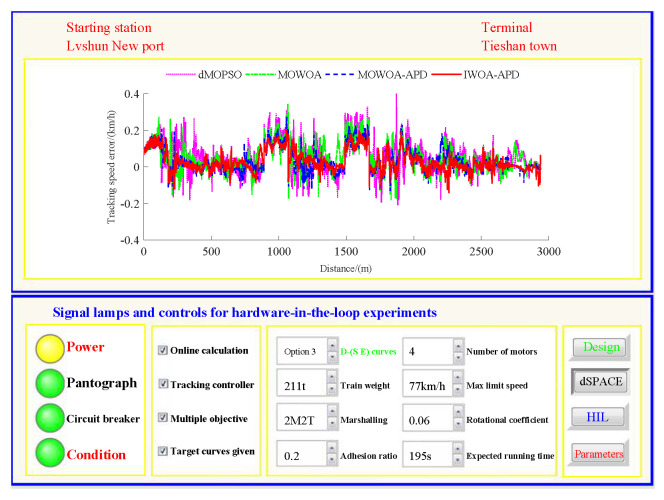
Tracking (speed error)–distance curves obtained by each optimization algorithm in Optimization Scenario 1.

**Figure 23:**
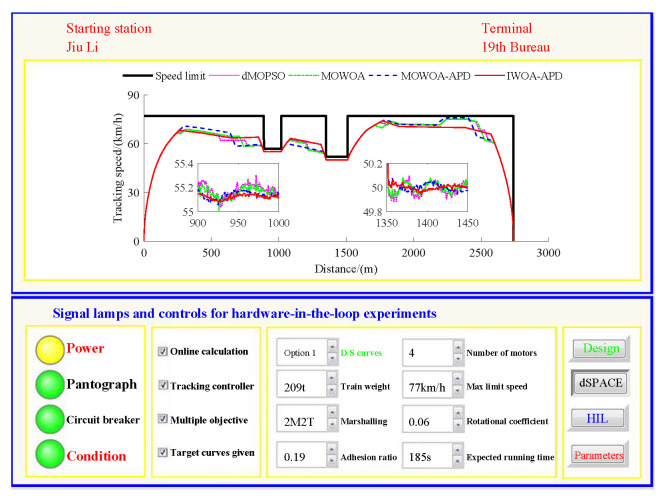
Tracking speed–distance curves obtained by each optimization algorithm in Optimization Scenario 2.

**Figure 24:**
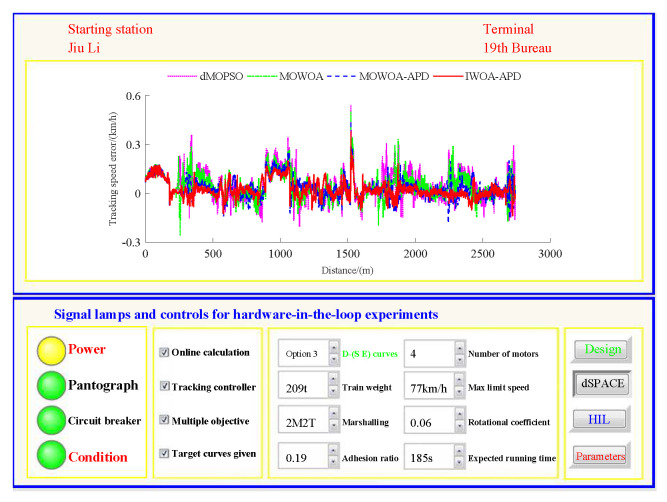
Tracking (speed error)–distance curves obtained by each optimization algorithm in Optimization Scenario 2.

On the premise of given target speed and ideal operating mode curves, it is necessary to realize high-quality tracking control for the target speed curve so as to finally obtain the comprehensive performance of ATO with low energy consumption, high comfort level, high punctuality, and accurate parking. The dynamic matrix control (DMC) algorithm is the first-generation model predictive control technology. Because of its strong control stability and good tracking control quality, DMC is widely used in the tracking control link of ATO [39]. To ensure fairness, this study adopts DMC with the same parameter settings for tracking control. The main parameter settings are as follows: the control period $T_{C}$ is 0.05 s, the modeling time domain length *N* is 60, the optimization time domain length *P* is 15, the control time domain length *L* is 15, and the softening factor α is 0.91. In order to further verify the effectiveness of the proposed IWOA-APD, the target speed and ideal operating mode curves are obtained through IWOA-APD, MOWOA-APD, MOWOA, and dMOPSO for Optimization Scenarios 1 and 2. Under the same HIL experimental environment for target speed curve tracking control, the DMC with the above parameter settings is adopted to implement the tracking control for various target speed curves [40]. The specific HIL test results for target speed tracking control (including tracking control speed–distance curves, tracking (speed error)–distance curves, and performance index tracking control results) are shown in Figure 21, Figure 22, Figure 23 and Figure 24, Table 11 and Table 12.

In Figure 21, Figure 22, Figure 23 and Figure 24, the power is turned on, the virtual pantograph module is in a normal state, the main circuit breaker is closed normally, and there is no abnormal situation in the HIL test environment for all target speed curves tracking control. According to Table 11 and Table 12, it can be seen that under the same HIL experimental environment of the target speed curve tracking control, IWOA-APD obtains the target velocity curve that is easier to track control. Compared with the multi-objective optimization algorithms MOWOA-APD, MOWOA, and dMOPSO used for comparative verification, it obtains significantly better performance index tracking control results (energy saving, punctuality, comfort level, and parking accuracy are all improved to a considerable extent). It can be seen from Figure 21, Figure 22, Figure 23 and Figure 24 that under the same HIL experimental environment of the target speed curve tracking control, compared with the multi-objective optimization algorithms MOWOA-APD, MOWOA, and dMOPSO used for comparison verification, IWOA-APD can obtain the target speed curve that is easier to track control. It can maintain the appropriate speed more smoothly, and hence it can suppress the speed fluctuation more effectively in the tracking control process, so the speed error is decreased sharply.

Further comparative explanation is as follows. As can be seen from Figure 15, Figure 16, Figure 18 and Figure 19, compared with IWOA-APD, traditional optimization methods (MOWOA and dMOPSO) and the improved optimization method (MOWOA-APD) do not have enough global optimization performance. To make sure that sufficiently small time and parking errors are obtained, it has to settle for the second best, so more complex ATO processes with several unnecessary operation sections are forced to be accepted and energy consumption and comfort level are also increased. It can be reflected from Figure 21, Figure 22, Figure 23 and Figure 24, compared to the IWOA-APD, that the urban rail train target speed curves obtained by traditional optimization methods (MOWOA and dMOPSO) and the improved optimization method (MOWOA-APD) is more difficult for tracking control.

## 6. Conclusions

In this study, an IWOA-APD for ATO is developed. Specifically, to solve the issue of traditional evaluation strategies, namely the disadvantages of inaccuracy, opacity and subject, a novel reasonable and applied comprehensive evaluation strategy is designed. In addition, an improved WOA is proposed based on strategies of non-linear decreasing convergence factor, solutions of out-of-bounds eliminating via combination of reflection and refraction, mechanisms of genetic evolution with variable probability, elite maintenance based on fusion distance, and crowding degree distance.

First of all, using angular penalty distance (APD) as an evaluation index can effectively and dynamically balance the importance of the convergence distribution during the evolution process so as to effectively improve the global optimization ability of the IWOA-APD. Second, an exponential non-linear decreasing strategy is adopted. By selecting the most appropriate optimization factor βα, the non-linear recursion can be optimized and adjusted. Utilizing the decreasing trend of the reduction function further enhances the global optimization ability of the IWOA-APD. Finally, by combining the fusion distance and the crowding distance, an elite set is constructed. Therefore, this optimization algorithm has a stronger ability to maintain population diversity, and it is possible to improve the global convergence ability of the IWOA-APD.

As can be seen, the simulation accomplishes optimization of about four benchmark functions. Compared with dMOPSO, MOWOA, and MOWOA-APD, the improved IWOA-APD enhances the global optimization quality excellently. Aiming at furtherly verifing the performance of the IWOA-APD proposed in this study, a HIL experimental platform for target speed curves optimization and tracking control is built. The test results verify the efficacy for IWOA-APD. The improved IWOA-APD achieves the advantages of fast computation speed, small time and parking errors, tiny comfort level measure value, and substantially decreased energy consumption.

## Figures and Tables

**Table 1:** Classification of quality evaluation levels for ATO of Dalian urban rail transit line 12 and line 13.

Performance Index	Excellent	Medium	Poor
Energy consumption	$\left(E_{0},E_{1}\right]$ kJ	$\left(E_{1},E_{2}\right]$ kJ	$\left(E_{2},+\infty \right)$ kJ
Punctuality	$\left(0,0.2\right]$ s	$\left(0.2,0.3\right]$ s	$\left(0.3,+\infty \right)$ s
Parking accuracy	$\left(0,0.1\right]$ m	$\left(0.1,0.2\right]$ m	$\left(0.2,+\infty \right)$ m
Comfort level	$\left(0,4.2\right]$ m/s^3^	$\left(4.2,7.5\right]$ m/s^3^	$\left(7.5,+\infty \right)$ m/s^3^

**Table 2:** Distance ranking of each elite in elite set.

Distance Ranking	Elite 1	Elite 2	Elite 3	Elite 4	Elite 5	Elite 6	Elite 7
Crowding degree distance ranking	5	1	4	3	1	5	6
Fusion distance ranking	2	1	5	4	3	1	2
Mean distance ranking	3.5	1	4.5	3.5	2	3	4
Distance comprehensive ranking	4	1	7	4	2	3	6

**Table 3:** The results about obtained complex tuning parameters of IWOA-APD in the ZDT1 scenario.

Parameter Name	Parameter Symbol	Value or Range
Variable rate factor	$\alpha_{P}$	1.68
Crossover probability	$\left[p_{c_{i},\min},p_{c_{i},\max}\right]$	$\left[0.65,0.85\right]$
Mutation probability	$\left[p_{m_{i},\min},p_{m_{i},\max}\right]$	$\left[0.06,0.09\right]$
Selection probability	$\left[p_{s_{i},\min},p_{s_{i},\max}\right]$	$\left[0.4,0.6\right]$
Ranges for the projection ratio	$\left[f_{\min},f_{\max}\right]$	$\left[1.5,3.5\right]$
Refractive index	$\left[{n_{r}}_{\min},{n_{r}}_{\max}\right]$	$\left[0.5,0.8\right]$
Attenuation coefficient	$\left[\beta_{\min,}\beta_{\max}\right]$	$\left[0.7,0.9\right]$

**Table 4:** IGD values obtained by each multi-objective optimization algorithm.

Optimization Algorithm	ZDT1	ZDT2	DTLZ1	DTLZ2
IWOA-APD	$5.44\times 10^{-4}$	$6.17\times 10^{-4}$	$2.74\times 10^{-3}$	$3.35\times 10^{-3}$
MOWOA-APD	$8.07\times 10^{-4}$	$1.06\times 10^{-3}$	$5.30\times 10^{-3}$	$5.49\times 10^{-3}$
MOWOA	$1.76\times 10^{-3}$	$1.85\times 10^{-3}$	$7.86\times 10^{-3}$	$1.03\times 10^{-2}$
dMOPSO	$2.28\times 10^{-3}$	$3.96\times 10^{-3}$	$1.33\times 10^{-2}$	$2.57\times 10^{-2}$

**Table 5:** Calculation time for each multi-objective optimization algorithm.

Optimization Algorithm	ZDT1	ZDT2	DTLZ1	DTLZ2
IWOA-APD	115.4 s	122.7 s	240.6 s	285.3 s
MOWOA-APD	154.9 s	160.9 s	297.1 s	366.5 s
MOWOA	158.7 s	163.0 s	302.8 s	369.1 s
dMOPSO	185.0 s	189.2 s	375.1 s	401.5 s

**Table 6:** Main parameters for Optimization Scenario 1.

Parameter Name (unit)	Parameter Value
Vehicle weight (t)	211
Maximum vehicle speed limit (km/h)	77
Distance between stations (m)	2940
Expected operating time (s)	195
Maximum allowable parking error (m)	0.2
Maximum allowable punctuality error (s)	0.3

**Table 7:** Main parameters for Optimization Scenario 2.

Parameter Name (unit)	Parameter Value
Vehicle weight (t)	209
Maximum vehicle speed limit (km/h)	77
Distance between stations (m)	2740
Expected operating time (s)	185
Maximum allowable parking error (m)	0.2
Maximum allowable punctuality error (s)	0.2

**Table 8:** Performance index optimization results obtained by each optimization algorithm in Optimization Scenario 1.

Optimization Algorithm	Energy Consumption	Time Error	Distance Error	Comfort Level
IWOA-APD	69,852 (kJ)	0.0257 (s)	0.0198 (m)	5.485 (m/s^2^/km)
MOWOA-APD	75,203 (kJ)	0.0574 (s)	0.0673 (m)	5.890 (m/s^2^/km)
MOWOA	79,874 (kJ)	0.0739 (s)	0.0844 (m)	6.527 (m/s^2^/km)
dMOPSO	82,390 (kJ)	0.1027 (s)	0.0942 (m)	6.845 (m/s^2^/km)

**Table 9:** Performance index optimization results obtained by each optimization algorithm in Optimization Scenario 2.

Optimization Algorithm	Energy Consumption	Time Error	Distance Error	Comfort Level
IWOA-APD	86,409 (kJ)	0.0493 (s)	0.0584 (m)	5.820 (m/s^2^/km)
MOWOA-APD	90,337 (kJ)	0.0751 (s)	0.0870 (m)	6.192 (m/s^2^/km)
MOWOA	93,085 (kJ)	0.0948 (s)	0.1058 (m)	6.385 (m/s^2^/km)
dMOPSO	95,330 (kJ)	0.1105 (s)	0.0977 (m)	6.547 (m/s^2^/km)

**Table 10:** Calculation time required for each optimization algorithm in Optimization Scenario 1 and Optimization Scenario 2.

Optimization Algorithm	Optimization Scenario 1	Optimization Scenario 2
IWOA-APD	904 s	873 s
MOWOA-APD	958 s	926 s
MOWOA	1082 s	1047 s
dMOPSO	1260 s	1193 s

**Table 11:** Performance index tracking control results obtained by each optimization algorithm in Optimization Scenario 1.

Optimization Algorithm	Energy Consumption	Time Error	Distance Error	Comfort Level
IWOA-APD	80,617 (kJ)	0.0486 (s)	0.0357 (m)	30.05 (m/s^2^/km)
MOWOA-APD	87,753 (kJ)	0.0833 (s)	0.0812 (m)	32.31 (m/s^2^/km)
MOWOA	95,284 (kJ)	0.1254 (s)	0.1087 (m)	33.86 (m/s^2^/km)
dMOPSO	99,650 (kJ)	0.1283 (s)	0.1160 (m)	35.27 (m/s^2^/km)

**Table 12:** Performance index tracking control results obtained by each optimization algorithm in Optimization Scenario 2.

Optimization Algorithm	Energy Consumption	Time Error	Distance Error (m)	Comfort Level
IWOA-APD	99,028 (kJ)	0.0775 (s)	0.0830 (m)	36.68 (m/s^2^/km)
MOWOA-APD	106,108 (kJ)	0.0924 (s)	0.0951 (m)	39.83 (m/s^2^/km)
MOWOA	115,292 (kJ)	0.1195 (s)	0.1270 (m)	41.25 (m/s^2^/km)
dMOPSO	118,386 (kJ)	0.1360 (s)	0.1178 (m)	42.90 (m/s^2^/km)

## Data Availability

The original contributions presented in this study are included in the article. Further inquiries can be directed to the corresponding author.

## References

[1] Liang Y., Liu H., Qian C., Wang G. (2019). A Modified Genetic Algorithm for Multi-Objective Optimization on Running Curve of Automatic Train Operation System Using Penalty Function Method. Int. J. Intell. Transp. Syst. Res..

[2] WWang P., Goverde R.M. (2016). Multiple-phase Train Trajectory Optimization with Signaling and Operational Constraints. Transp. Res. Part C Emerg. Technol..

[3] Huang D., Yi S., Li X. (2022). Research on Accurate Parking Control for Urban Rail Trains via Robust Adaptive Backstepping Approach. IEEE Trans. Intell. Transp. Syst..

[4] Khmelnitsky E. (2000). On an optimal control problem of train operation. IEEE Trans. Autom. Control.

[5] Chang C., Sim S. (1997). Optimising train movements through coast control using genetic algorithms. Proc. Electr. Power Appl..

[6] Fernandez-Rodr1’guez A., Fernandez-Cardador A., Cucala A.P., Dominguez M., Gonsalves T. (2015). Design of robust and energy-efficient ATO speed profiles of metropolitan lines considering train load variations and delays. IEEE Trans. Autom. Sci. Eng..

[7] Wei S.G., Yan X.H., Cai B.G., Wang J. (2015). Multiobjective optimization for train speed trajectory in CTCS high-speed railway with hybrid evolutionary algorithm. IEEE Trans. Intell. Transp. Syst..

[8] Gu Q., Tang T., Cao F., Song Y.-D. (2014). Energy-Efficient Train Operation in Urban Rail Transit Using Real-Time Traffic Information. IEEE Trans. Intell. Transp. Syst..

[9] Gu Q., Tang T., Ma F. (2016). Energy-Efficient Train Tracking Operation Based on Multiple Optimization Models. IEEE Trans. Intell. Transp. Syst..

[10] Yin J., Chen D., Li L. (2014). Intelligent Train Operation Algorithms for Subway by Expert System and Reinforcement Learning. IEEE Trans. Intell. Transp. Syst..

[11] Cheng R., Yu W., Song Y., Chen D., Ma X., Cheng Y. (2019). Intelligent Safe Driving Methods Based on Hybrid Automata and Ensemble CART Algorithms for Multihigh-Speed Trains. IEEE Trans. Cybern..

[12] Li X., Wu H., Yang Q., Tan S., Xue P., Yang X. (2022). A multistrategy hybrid adaptive whale optimization algorithm. J. Comput. Des. Eng..

[13] Li M., Xu G., Zeng L., Lai Q. (2023). Hybrid whale optimization algorithm based on symbiosis strategy for global optimization. Appl. Intell..

[14] Li M., Xu G., Zeng L., Lai Q. (2020). New binary whale optimization algorithm for discrete optimization problems. Eng. Optim..

[15] Motwani A., Shukla P.K., Pawar M., Arya M., Jain P. (2024). Deep-CNWO: A deep-chaotic nature whale optimization algorithm for early prediction of blood pressure disorder in smart healthcare settings. Neural Comput. Appl..

[16] Hassan A.A., Abdullah S., Zamli K.Z., Razali R. (2023). Q-learning whale optimization algorithm for test suite generation with constraints support. Neural Comput. Appl..

[17] Fan Q., Chen Z., Zhang W., Fang X. (2022). Engineering with Computers ESSAWOA: Enhanced Whale Optimization Algorithm integrated with Salp Swarm Algorithm for global optimization. Eng. Comput..

[18] Song H., ShangGuan W., Qiu W., Sheng Z., Harrod S.S. (2023). Two-Stage Optimal Trajectory Planning Based on Resilience Adjustment 664 Model for Virtually Coupled Trains. IEEE Trans. Intell. Transp. Syst..

[19] Miyatake M., Ko H. (2010). Optimization of train speed profile for minimum energy consumption. IEEJ Trans. Electr. Electron. Eng..

[20] Zhong W., Li S., Xu H., Zhang W. (2010). On-Line Train Speed Profile Generation of High-Speed Railway With Energy-Saving: A Model Predictive Control Method. IEEE Trans. Intell. Transp. Syst..

[21] Said L.B., Bechikh S., Dira K. (2010). The rdominance: A new dominance relation for interactive evolutionary multicriteria decision making. IEEE Trans. Evol. Comput..

[22] Talbi E.G. (2009). Metaheuristics: From Design to Implementation.

[23] Cheng R., Jin Y., Olhofer M., Sendhoff B. (2016). A reference vector guided evolutionary algorithm for many objective optimization. IEEE Trans. Evol. Comput..

[24] Aziz M.A.E., Ewees A.A., Hassanien A.E. (2017). Whale Optimization Algorithm and Moth-Flame Optimization for multilevel thresholding image segmentation. Expert Syst. Appl..

[25] Kaur G., Arora S. (2018). Chaotic Whale Optimization Algorithm. J. Comput. Des. Eng..

[26] Parsopoulos K.E., Vrahatis M.N. (2002). Recent approaches to global optimization problems through Particle Swarm Optimization. Nat. Comput..

[27] Tizhoosh H.R. Opposition-Based Learning: A New Scheme for Machine Intelligence. Proceedings of the International Conference on Computational Intelligence for Modelling, Control Automation, International Conference on Intelligent Agents, Web Technologies Internet Commerce.

[28] Zhang H.P. (2021). On the convergence of a cooperative bat searching algorithm. Eur. J. Control.

[29] Prasad D., Mukherjee A., Shankar G., Mukherjee V. (2006). Application of chaotic whale optimization algorithm for transient stability constrained optimal power flow. IET Sci. Meas. Technol..

[30] Liu G., Wang X. Fault diagnosis of diesel engine based on fusion distance calculation. Proceedings of the Advanced Information Management, Communicates, Electronic Automation Control Conference.

[31] Liu G., Zhou X., Xu X., Wang L., Zhang W. (2022). Fault Diagnosis of Diesel Engine Information Fusion based on Adaptive Dynamic Weighted Hybrid Distance-Taguchi method (ADWHD-T). Appl. Intell..

[32] Liu G., Xu C., Wang L. (2023). Modified ADRC Design of Permanent Magnet Synchronous Motor Based on Improved Memetic Algorithm. Sensors.

[33] Peng H., Li R., Cao L., Li L. (2011). Multiple Swarms Multi-Objective Particle Swarm Optimization Based on Decomposition. Procedia Eng..

[34] Abd El Aziz M., Ewees A.A., Hassanien A.E. (2018). Multi-objective whale optimization algorithm for content-based image retrieval. Multimed. Tools Appl..

[35] Deb K., Thiele L., Laumanns M. (2002). Zitzler, E. Scalable multi-objective optimization test problems. IEEE.

[36] Zhang H., Zhang Y., Yin C. (2016). Hardware-in-the-Loop Simulation of Robust Mode Transition Control for aSeries-Parallel Hvbrid Electric Vehicle. EEE Trans. Veh. Technol..

[37] Yu S., Han J., Qu Z., Yang Y. (2018). A Force and Displacement Compensation Method Toward Divergenceand Accuracy of Hardware-in-the-Loop Simulation Svstem for Manipulator Docking. IEEE Access.

[38] Xi G., Zhao X., Liu Y., Huang J., Deng Y. (2019). A hierarchical ensemble learning framework for energy-efficient automatic train driving. Tsinghua Sci. Technol..

[39] Mi X., Zou Y., Li S., Karimi H. (2020). Self-triggered DMPc Design for Cooperative Multi-agent Systems. IEEE Trans. Ind. Inform..

[40] Wang L., Cheng Y., Zou J. (2014). Battery available power prediction of hybrid electric vehicle based on improvedWnamic Matrix Control algorithms. Power Sources.

